# Cell identity dynamics and insight into insulin secretagogues when employing stem cell-derived islets for disease modeling

**DOI:** 10.3389/fbioe.2024.1392575

**Published:** 2024-06-12

**Authors:** Chencheng Wang, Shadab Abadpour, Aleksandra Aizenshtadt, Andrea Dalmao-Fernandez, Merete Høyem, Ingrid Wilhelmsen, Justyna Stokowiec, Petter Angell Olsen, Stefan Krauss, Simona Chera, Luiza Ghila, Helge Ræder, Hanne Scholz

**Affiliations:** ^1^ Department of Transplant Medicine and Institute for Surgical Research, Oslo University Hospital, Oslo, Norway; ^2^ Hybrid Technology Hub, Center of Excellence, University of Oslo, Oslo, Norway; ^3^ Section for Pharmacology and Pharmaceutical Biosciences, Department of Pharmacy, University of Oslo, Oslo, Norway; ^4^ Department of Immunology and Transfusion Medicine, Oslo University Hospital, Oslo, Norway; ^5^ Department of Clinical Science, University of Bergen, Bergen, Norway; ^6^ Department of Pediatrics, Haukeland University Hospital, Bergen, Norway

**Keywords:** stem cell derived beta cells, insulin secretagogues, disease modeling, pyruvate kinase (pk), tri-hormonal cells

## Abstract

Stem cell-derived islets (SC-islets) are not only an unlimited source for cell-based therapy of type 1 diabetes but have also emerged as an attractive material for modeling diabetes and conducting screening for treatment options. Prior to SC-islets becoming the established standard for disease modeling and drug development, it is essential to understand their response to various nutrient sources *in vitro*. This study demonstrates an enhanced efficiency of pancreatic endocrine cell differentiation through the incorporation of WNT signaling inhibition following the definitive endoderm stage. We have identified a tri-hormonal cell population within SC-islets, which undergoes reduction concurrent with the emergence of elevated numbers of glucagon-positive cells during extended *in vitro* culture. Over a 6-week period of *in vitro* culture, the SC-islets consistently demonstrated robust insulin secretion in response to glucose stimulation. Moreover, they manifested diverse reactivity patterns when exposed to distinct nutrient sources and exhibited deviant glycolytic metabolic characteristics in comparison to human primary islets. Although the SC-islets demonstrated an aberrant glucose metabolism trafficking, the evaluation of a potential antidiabetic drug, pyruvate kinase agonist known as TEPP46, significantly improved *in vitro* insulin secretion of SC-islets. Overall, this study provided cell identity dynamics investigation of SC-islets during prolonged culturing *in vitro*, and insights into insulin secretagogues. Associated advantages and limitations were discussed when employing SC-islets for disease modeling.

## Introduction

Insulin is a critical regulator of energy metabolism, which directs the usage of carbohydrates, fats, and proteins throughout the body (Hogrebe et al., 2023). Exogenous insulin injections have been a primary treatment for both type 1 (T1D) and severe type 2 diabetes (T2D)(Salib et al., 2022). Although exogenous insulin injection partially compensates for the role of endogenous insulin, continuous, rapid, and accurate energy metabolism regulation by healthy beta cells is missing. In addition, the root causes of diabetes remain unsolved. Evidence increasingly shows that T1D and T2D are pathogenically heterogeneous (Balboa et al., 2019; Bar-Tana, 2020). Thus, reliable diabetes models are needed for developing efficient therapies.

Human beta cell lines like the EndoC-βH1 cell line can provide valuable insights into beta cell physiology and pathophysiology. Yet the beta cell line alone is unable to mimic the physiological regulation networks and the cell-cell interactions within the islets (Walker et al., 2021). Animal models including rodents and porcine and their isolated islets are remarkably helpful for studying biological processes. However, they often respond to experimental interventions in ways that differ strikingly from humans because of species differences (Cefalu, 2006; Balboa et al., 2019). Primary human islets isolated from deceased pancreas donors provide invaluable experimental material. However, its widespread usage is hindered by limited availability, donor variability, relative short viability in culture, heterogeneity, and ethical and regulatory considerations (Mohandas et al., 2023). Pancreatic tissue slices enable study of human islet cell physiology at the cellular to multi-islet level. However, its limited for short-term applications (Qadir et al., 2020; Cohrs et al., 2023). In 2023, the U.S. Food and Drug Administration abolished the mandate for animal testing as a prerequisite for human trials for all drugs (Wadman, 2023). In addition, stem cell-derived beta cells (SC-islets) that secreted insulin in response to glucose stimulation were able to be generated and consistently improved *in vitro* (Pagliuca et al., 2014; Rezania et al., 2014; Barsby and Otonkoski, 2022). Consequently, emerging technology such as “organoids” and “organ-on-chips” acts as promising new tools for drug testing.

Although these protocols generate functional insulin-producing cells, there is still low differentiation efficiency and low maturity in addition to off-target cells that need to be improved. It has been shown that endocrine cell fate selection and maturation can be improved by modulation of the WNT signaling at various differentiation stages (Mahaddalkar et al., 2020; Böttcher et al., 2021; Katsumoto et al., 2022). One example is the treatment with IWP2, a small-molecule inhibitor of WNT ligand secretion after the definitive endoderm (DE) stage promotes pancreatic cell differentiation (Sharon et al., 2019b). In addition, inhibition of the WNT signaling after the pancreatic progenitor (PP) stage improves the endocrine lineage specification (Vethe et al., 2019). Therefore, we used a recently established two-dimensional (2D) differentiation protocol for generating SC-islets (Hogrebe et al., 2020; 2023), and investigated whether manipulating the WNT signaling pathway on top of this 2D differentiation protocol could further improve the efficiency.

Furthermore, to accurately reflect the physiology of human islets, it is crucial to meticulously compare the utilization and responses of SC-islets to various nutrients with those of human primary islets (Beydag-Tasöz et al., 2023; Jensen and Little, 2023). For example, we have coupled SC-islets and SC-liver organoids in a recirculating organ-on-chip (rOoC) platform and generated a metabolic crosstalk model between islets and liver (Aizenshtadt et al., 2024). To provide insights before SC-islets spread into downstream utilization for drug testing and development, this study developed a merged protocol based on recently developed SC-islets differentiation protocols (Hogrebe et al., 2020; 2021; Mahaddalkar et al., 2020). This merged protocol offers a flexible and relatively low-cost laboratory-scale manufacturing process. The assessments on SC-islets in terms of *in vitro* and *in vivo* function analysis were performed, with an additional focus on assessing different insulin secretagogues. The metabolism gap between SC-islets and human primary islets was analyzed. A pharmacological compound, TEPP46, which can activate pyruvate kinase and is known for enhancing insulin secretion (Abulizi et al., 2020; Lewandowski et al., 2020), was tested on the SC-islets to evaluate potential antidiabetic drugs.

## Results

### WNT inhibition after definitive endoderm stage enhanced pancreatic endocrine cell commitment in planar differentiation protocol

**FIGURE 1:**
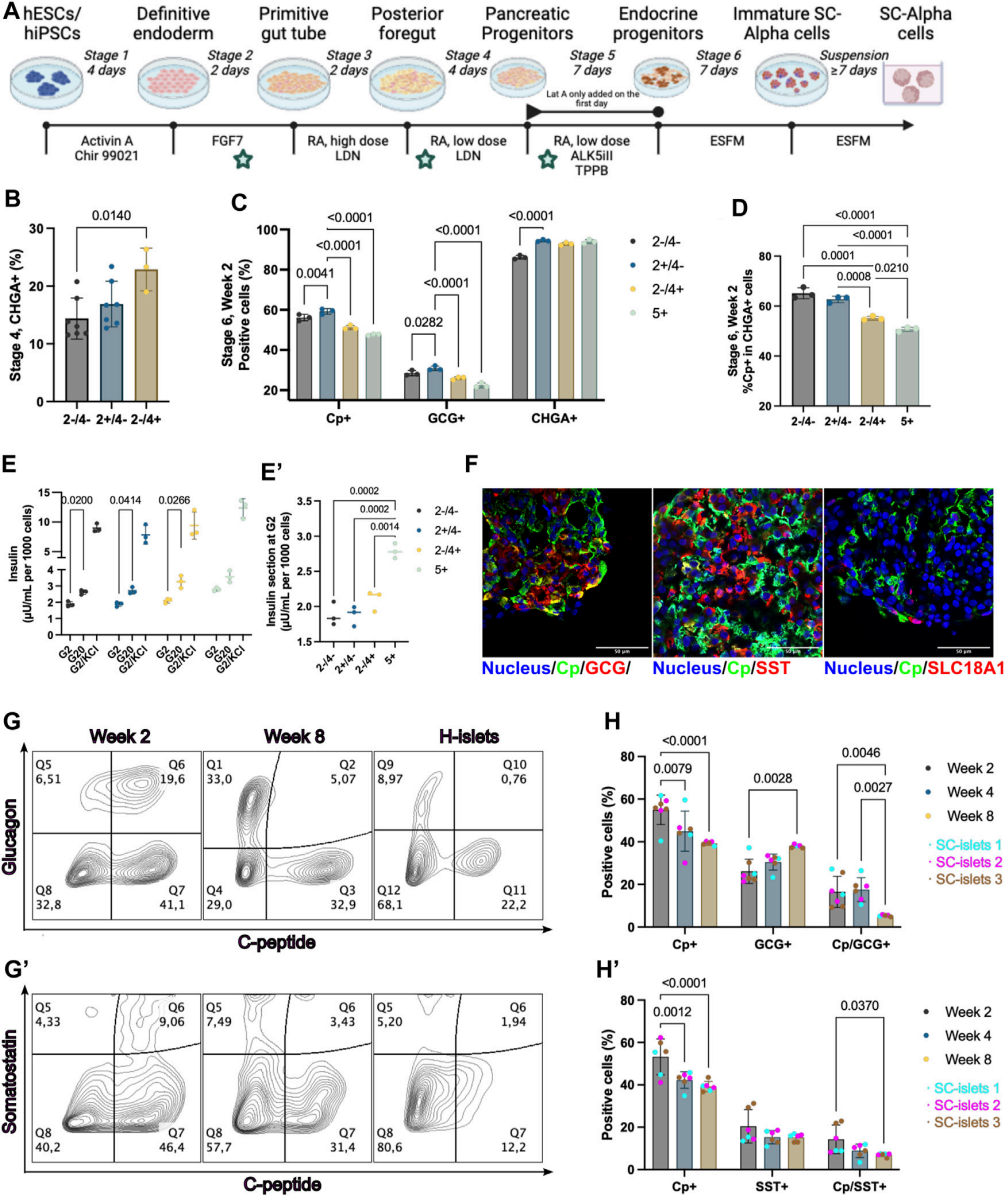
WNT inhibition at stage 2 enhanced pancreatic endocrine cell commitment. **(A)** Schematic depicting the 2D differentiation protocol used in this study. The time points of WNT inhibition by IWP2 were labeled with a pentagram. **(B)** Flow cytometry quantification of CHGA + cells at stage 4. “+”, IWP2 added; “−” without IWP2. Statistical test: One-way ANOVA with Turkey’s multiple comparisons test. *n* = 3–7. **(C)** Flow cytometry quantification of Cp+, GCG+, and CHGA + cells at week 2 of stage 6. Statistical test: Two-way ANOVA with Dunnett’s multiple comparisons test. Group “2+/4-” was taken as the control group. n = 3. **(D)** Flow cytometry quantification of Cp + cells percentage among CHGA + cells at week 2 of stage 6. Statistical test: One-way ANOVA with Turkey’s multiple comparisons test. *n* = 3. **(E)** Static GSIS of SC-islets generated under WNT inhibition at different stages. Arabic numeral representing the stages of differentiation; “+”, IWP2 added; “−” without IWP2. Statistical test: Two-way ANOVA with Dunnett’s multiple comparisons test (G2 V s. G20). N = 3. **(E′)** Insulin secretion at 2 mM glucose concentration during static GSIS from different groups. Statistical test: One-way ANOVA with Turkey’s multiple comparisons test. N = 3. **(F)** Representative immunostaining sections for SC-islets markers and SC-EC cell markers. Scale bar = 50 µm. **(G and G′)** Representative flow cytometry plots of SC-islets and human islets (H-islets) after prolonged culturing *in vitro*. **(H and H′)** Flow cytometry quantification for Cp, GCG, and SST of SC-islets after prolonged culturing *in vitro*. Statistical test: Two-way ANOVA with Turkey’s multiple comparisons test. *n* = 3, N = 2. CP, C-Peptide; GCG, glucagon, SST, Somatostatin; CHGA, Chromogranin A; SLC18A1, Vesicular monoamine transporter 1; Nucleus stained with Hoechst 33342.

The DE differentiation stage varies from 3- to 4 days in different studies (Sharon et al., 2019a; Hogrebe et al., 2021). To confirm the suitable duration of differentiation to DE cells, we incubated the cells for 3- or 4-day in stage 1 and found no significant difference in differentiation efficiency (Supplementary Figure S1A). Thus, we have chosen the 4-day incubation at stage 1 to align with the backbone 2D protocol we were based on (Hogrebe et al., 2020). IWP2 was introduced at stages 2, 4, and 5, respectively (Figure 1A). The WNT inhibition at stage 2 by IWP2 did not significantly affect PDX1+ cell commitment (Supplementary Figure S1B). In addition, the WNT inhibition at stage 2 or 4 did not substantially affect the PP cell differentiation, which was evaluated by analyzing the PDX1+/NKX6.1+ cell population (Supplementary Figure S1C). Chromogranin A (CHGA), a pan-endocrine cell marker, was detectable at stage 4, and the WNT inhibition at stage 4 but not at stage 2 significantly increased the CHGA + cell population (Figure 1B, Supplementary Figure S1D). However, the NKX6.1 and CHGA co-expression was rarely detectable by immunofluorescence staining and flow cytometry analysis (Supplementary Figures S1D, E), depicting more non-beta progenitor cells’ introduction due to a WNT inhibition at stage 4 but not at stage 2.

It has been reported that WNT inhibition using IWR at stage 5 in 3D culture significant increases CHGA+ and C-peptide/NKX6.1+ cells differentiation (Sharon et al., 2019b). To investigate the effect of WNT inhibition at stage 5 current in our 2D differentiation protocol, we added IWP2 at stage 5. There was no significant difference at stage 5 on cells positive for C-peptide (Cp), Glucagon (GCG), and CHGA, nor when we calculated the percentage of Cp + cells among CHGA + cells at stage 5 (Supplementary Figures S1G, H). Moreover, the WNT inhibition by IWP2 at late stages (stage 4 or 5) induced a reduction in Cp+ and GCG + cell percentages observed among the final products at stage 6, and a reduced Cp+/CHGA + ratio (Figures 1C,D,F). The difference compared to the previous observation of WNT inhibition at stage 5 may be due to the 2D *versus* 3D culture systems used, as the study shows that the spatial environments can affect the SC-islets differentiation (Sharon et al., 2019b; Carrasco et al., 2022). Thus, our data showed that the WNT inhibition at stage 2, but not at the late stages (stage 4 or 5), generates the highest CHGA + cells while not significantly decreasing Cp + cell yielding.

To evaluate the effect of the WNT inhibition at stages 2, 4, or 5 on the functionality of the SC-islets at stage 6, a static glucose-stimulated insulin secretion (GSIS) measurement was performed respectively. All the SC-islets generated under different conditions showed a significant increase in insulin secretion in response to glucose (Figure 1E). However, the SC-islets with WNT inhibition at stage 5 showed a higher background insulin secretion in 2 mM glucose Krebs buffer (Krb) than those with or without WNT inhibition at stage 2 (Figure 1E’). In summary, our data showed that WNT inhibition at stage 2 compared to untreated or WNT inhibition at stage 4 or 5, enhanced pancreatic endocrine cell commitment without dismissing the Cp + cell’s differentiation efficiency and insulin secretion ability. Therefore, the subsequent investigation was conducted with the SC-islets generated with IWP2 treatment at stage 2.

### Bi-hormonal cell proportion decreased under long-term culturing *in vitro*

Within the SC-islets, C-peptide-positive cells constituted a majority, exceeding 50% (Figure 1C). Small amounts of unwanted cell populations such as bi-hormonal cells that are either Cp+/GCG+, Cp+/Somatostatin (SST)+ (Hogrebe et al., 2020; Mahaddalkar et al., 2020), or enterochromaffin-like cells (SC-ECs) represent mis-differentiated byproducts from SC-islets differentiation (Balboa et al., 2022; Sean et al., 2022). To evaluate the undesired poly-hormonal and off-target cells among SC-islets, we found that Cp+/GCG+ and Cp+/SST + cells still exist in the current products at stage 6, day 14 (Figure 1F). SC-ECs (SLC18A1+) were detectable (Figure 1F). In alignment with previous publications (Hogrebe et al., 2020; Mahaddalkar et al., 2020), the SC-islets express core identity markers, including CHAG, CXCR4, PDX1, NKX6.1, MAFA, and MAFB, similar to human islets (Supplementary Figure S2).

Prolonged SC-islet culture is often needed for disease modeling or drug evaluation (Bauer et al., 2017). To this end, we extended the culture for stage 6 SC-islets to 56 days (8 weeks) *in vitro* without supplements other than culturing in a defined enriched serum-free medium (ESFM). We observed the GCG+/Cp+ and SST+/Cp + bi-hormonal and the Cp + cell population decreased, whereas the GCG+/Cp- and SST+/Cp-single hormonal cell population increased from week 2–8 (Figure 1G, G’). Flow cytometry quantification confirmed a significant drop of bi-hormonal cells from week 2–8, for Cp+/GCG+ from 16.6% to 5.3%, and for Cp+/SST+ from 12.7% to 7.0% (Figure 1H, H’). In addition, the total GCG + cells significantly increased but not the SST + cells under prolonged culture *in vitro*. The total Cp + cells significantly declined simultaneously, whereas the CHGA + cells remained unchanged (Figure 1H, H’, Supplementary Figures S3A, B). These results provide provide quantitative evidence that the Cp+/GCG + bi-hormonal cells are more likely to develop as GCG + cells under prolonged culture *in vitro*.

### A tri-hormonal cell population decreased under prolonged culturing *in vitro*

**FIGURE 2:**
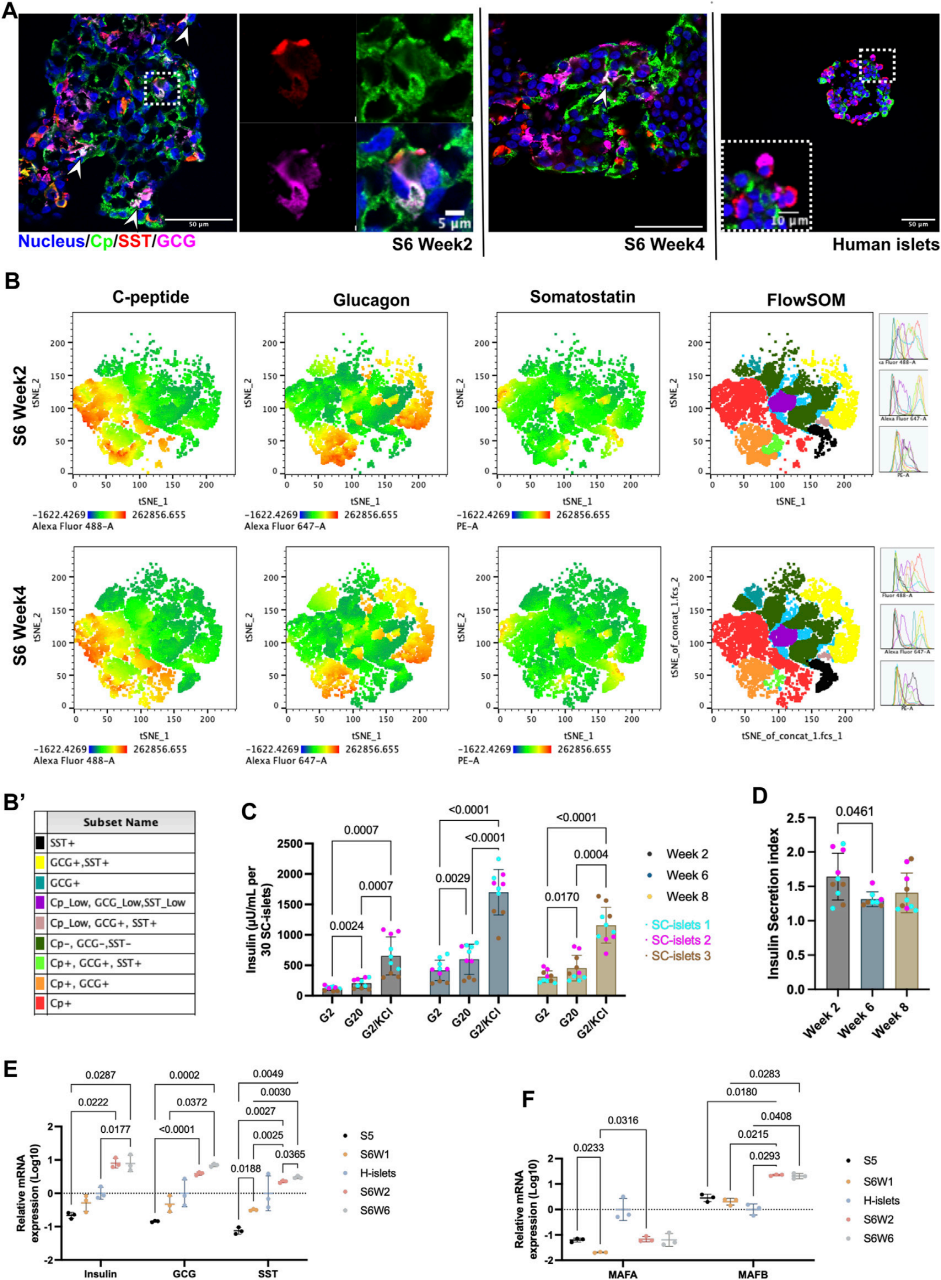
SC-islets under prolonged culturing *in vitro*. **(A)** Representative immunostaining sections for SC-islets and human islets for Cp, SST, and GCG. White arrowheads represent the tri-hormonal cells. **(B)** tSNE analysis of flow cytometry data for SC-islets under prolonged culturing in weeks 2 and 4. **(B′)** Sub-cell populations that identified with FlowSOM were labeled manually and annotated with different colors. **(C)** Static GSIS of the SC-islets under prolonged culturing *in vitro* in week 2/6/8 of stage 6. Statistical test: Two-way ANOVA with Turkey’s multiple comparisons test. *n* = 3, with N = 3–4. **(D)** Insulin secretion index of the SC-islets under prolonged culturing *in vitro* in week 2/6/8 of stage 6. Statistical test: One-way ANOVA with Turkey’s multiple comparisons test. n = 3, N = 2–4. **(E)** RT-qPCR quantification of human islets and SC-islets for *Insulin*, *GCG*, and *SST*. Statistical test: Two-way ANOVA with Turkey’s multiple comparisons test. n = 3. **(F)** RT-qPCR quantification of human islets and SC-islets for *MAFA* and *MAFB*. Statistical test: Two-way ANOVA with Turkey’s multiple comparisons test. *n* = 3. S5, stage 5; S6W1/2/6, week 1/2/6 of stage 6; H-islets, human islets. Statistical test: Two-way ANOVA with Turkey’s multiple comparisons test. *n* = 3 biological duplicates.

To further understand the details of SC-islets’ single hormonal cell commitment, we performed immunofluorescence staining with Cp, GCG, and SST antibodies. We observed a tri-hormonal cell population that was positive for Cp, GCG, and SST at week 2 of stage 6, and this tri-hormonal cell population was still observable but was less in week 4 of stage 6, as indicated with white arrowheads. (Figure 2A). The flow cytometry data supported the presence of SST + cells among Cp+/GCG + cell subpopulation (Supplementary Figure S3C). Of note, this tri-hormonal cells were also detectable in the control group that without IWP2 treatment at stage 2 (Supplementary Figure S3D). In addition, Cell populations identified with T-Distributed Stochastic Neighbor Embedding (tSNE) and FlowSOM were labeled and manually annotated, with special interests where populations labeled in purple and green shows the tri-hormonal cells (Figure 2B, B’, Supplementary Figure S3E, E’). In which these two clusters represents tri-hormonal cells that is positive for Cp, GCG and SST. The tri-hormonal cells were fuether characterized as GCG high, Cp/SST low population (Pop1, Supplementary Figure S3F) and SST high, Cp/GCG low population (Pop4, Supplementary Figure S3F) with FlowSOM analysis. From week 2–4 of stage 6, the number of tri-hormonal cells consistently decreased from 10% to less than 5%, followed by less than 3% in week 8 (Supplementary Figure S3G).

### SC-islets maintained their function for up to 8 weeks *in vitro*

To investigate the functionality of SC-islets under long-term culturing *in vitro*, we monitored the GSIS ability at stage 6 days 14 (week 2), day 42 (week 6), and day 56 (week 8) from three independent batches of differentiation. Three independent batches of SC-islets showed a significant increase in insulin secretion in response to 20 mM glucose stimulation from week 2 to week 8 (Figure 2C). We observed a slight but significant decrease in the insulin stimulation index (SI, insulin secreted in high glucose or indecated insulin secretagogues/insulin produced in low glucose) from week 2 to week 6, however, the SI is still maintained at 1.4 at week 8 (Figure 2D). GSIS analysis in week 2 of stage 6 is the most common timepoint selected for quality assessment in use of SC-islets differentiation protocols (Mahaddalkar et al., 2020; Hogrebe et al., 2021; Balboa et al., 2022). Although under debate, the SI > 1, determined from the ratio of insulin produced in high- and low-glucose, is currently a fundamental release criterion for human islets transplantation (Glieberman et al., 2021; Molano et al., 2024). That said, our data supported that the SC-islets can be used for downstream application in a 6-week time window (from weeks 2–8 of stage 6) without significant abnormality in GSIS ability. This time window provides flexibility for downstream interpretation of disease modeling.

To investigate the connections of gene expression to the GSIS function changes, we conducted the gene expression analysis under prolonged culture condition *in vitro*. The expressions of genes including, *Insulin*, *PDX1, NKX6.1, NKX2.2, MAFB, ISL1, NEUROD1* and *ABCC8,* were higher compared to human islets, while the disallowed gene such as *LDHA* is significantly lower (Supplementary Figure S3H). The *Insulin* expression was significantly increased in week 2 compared to week 1 of at stage 6 but remained unchanged under prolonged culturing (stage 6 weeks 2 vs. 6, Figure 2E). However, the *GCG* and *SST* expression was significantly increased under prolonged culturing in week 6 compared to week 2 at stage 6 (Figure 2E). The increase of *GCG* and *SST* gene expressions further indicated that the tri-hormonal and bi-hormonal cells may not be as likely to develop into Cp + cells under the current protocol.

The *MAFA* expression in SC-islets at week 2 of stage 6 was significantly lower than in human islets, with a downregulation from stage 5–6 during the differentiation (Figure 2F, Supplementary Figure S3H). However, under prolonged culture, the *MAFA* and *MAFB* mRNA expression levels remain unchanged (Figure 2F). MAFA was located in the nucleus at stages 5 and the first week of stage 6 (Supplementary Figure S4). However, under prolonged culture *in vitro*, the MAFA’s nucleus localization became less pronounced, whereas the MAFB was presented in nucleus (Supplementary Figure S4). Evidence supports that *MAFA* is a maturation marker for beta cells and keeps upregulation postnatal, while *MAFB* keeps downregulation (Balboa et al., 2022). Thus, the loss of MAFA nucleus localization may relate to the fact that SC-islets could not maintain their GSIS responses for more than 6 weeks *in vitro*.

### Transplantation of SC-islets prevents alloxan-induced diabetes in mice

**FIGURE 3:**
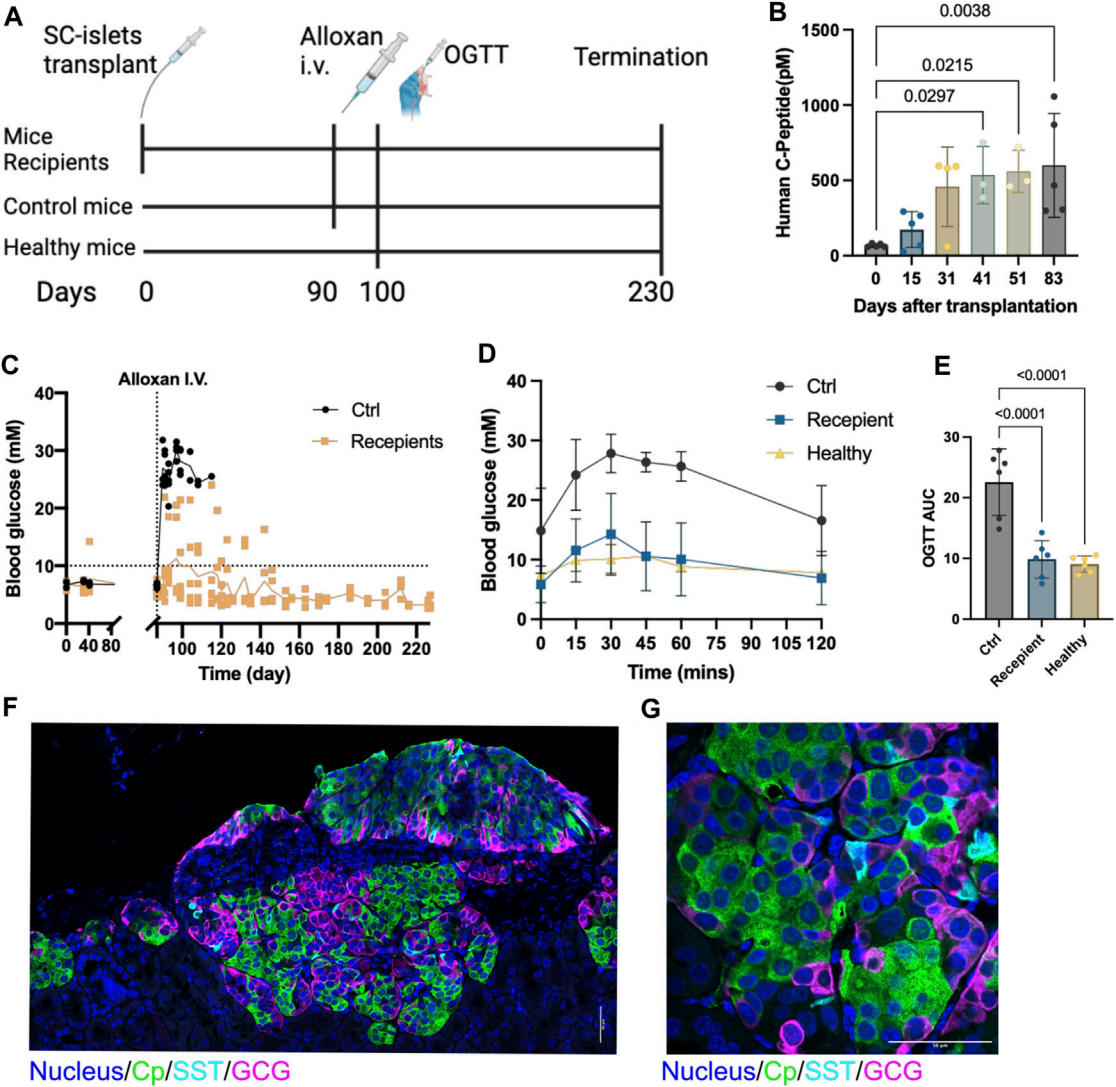
SC-islets transplantation prevents alloxan-induced diabetes in mice. **(A)** Experiments design diagram. **(B)** Human c-peptide detection in mice blood samples. Data presented as mean ± SD. Statistical test: One-way ANOVA with Dunnett’s multiple comparisons test. *n* = 5. **(C)** Blood glucose measurements before and after alloxan treatment and kidney capsule (KC) transplantation. The dotted line represents blood glucose at 10mM, the solid line represents the mean value of blood glucose. “Ctrl”, control. *n* = 5. **(D)** Oral glucose tolerance test (OGTT) blood glucose measurements. Ctrl: *n* = 2, Recipient: *n* = 5, Healthy: *n* = 2. **(E)** AUC quantification for OGTT, comparing six measured time points between 0 and 120 min post injection of each group. Statistical test: One-way ANOVA with Dunnett’s multiple comparisons test. **(F)** Immunostaining of sectioned kidneys transplanted with SC-islets 120 days after transplantation showing endocrine cell markers. **(G)** Immunostaining imaging of sectioned kidneys with a higher magnification showing endocrine cell markers. Cp, c-peptide; SST, somatostatin, GCG, glucagon. Nucleus stained with Hoechst 33432. Scale bar = 50 µm.

To evaluate the *in vivo* function, we transplanted two batches of the SC-islets (week 2 of stage 6) under the kidney capsule in 5 healthy male Rag1^−/−^ mice that do not contain mature B and T lymphocyte (Figure 3A). The human Cp was detectable at 2 weeks post-transplantation and increased from day 41 to day 83 post-transplantation (Figure 3B). Alloxan that ablates selectively rodent but not human beta cells was administrated at day 90 post-transplantation to induce diabetic mice. After Alloxan-induced diabetes, the random blood glucose was maintained at a normal range (<11 mM) in the mice with SC-islets grafts compared to control mice without SC-islets (Figure 3C). Furthermore, the oral glucose tolerance test (OGTT) at day 100 showed SC-islets grafted mice had a glucose tolerance similar to the healthy mice (Figure 3D), as well as when calculated as the area under curve (AUC) (Figure 3E). Immunostaining of the retrieved kidney with SC-islets grafts after termination of the studies revealed a large region of Cp+ and GCG + cells, with a few SST + cells presented (Figures 3F,G). These data demonstrated that these SC-islets prevented Alloxan-induced diabetes in mice.

### Insulin secretion from SC-islets in response to different nutrients was differed from human primary islets

**FIGURE 4:**
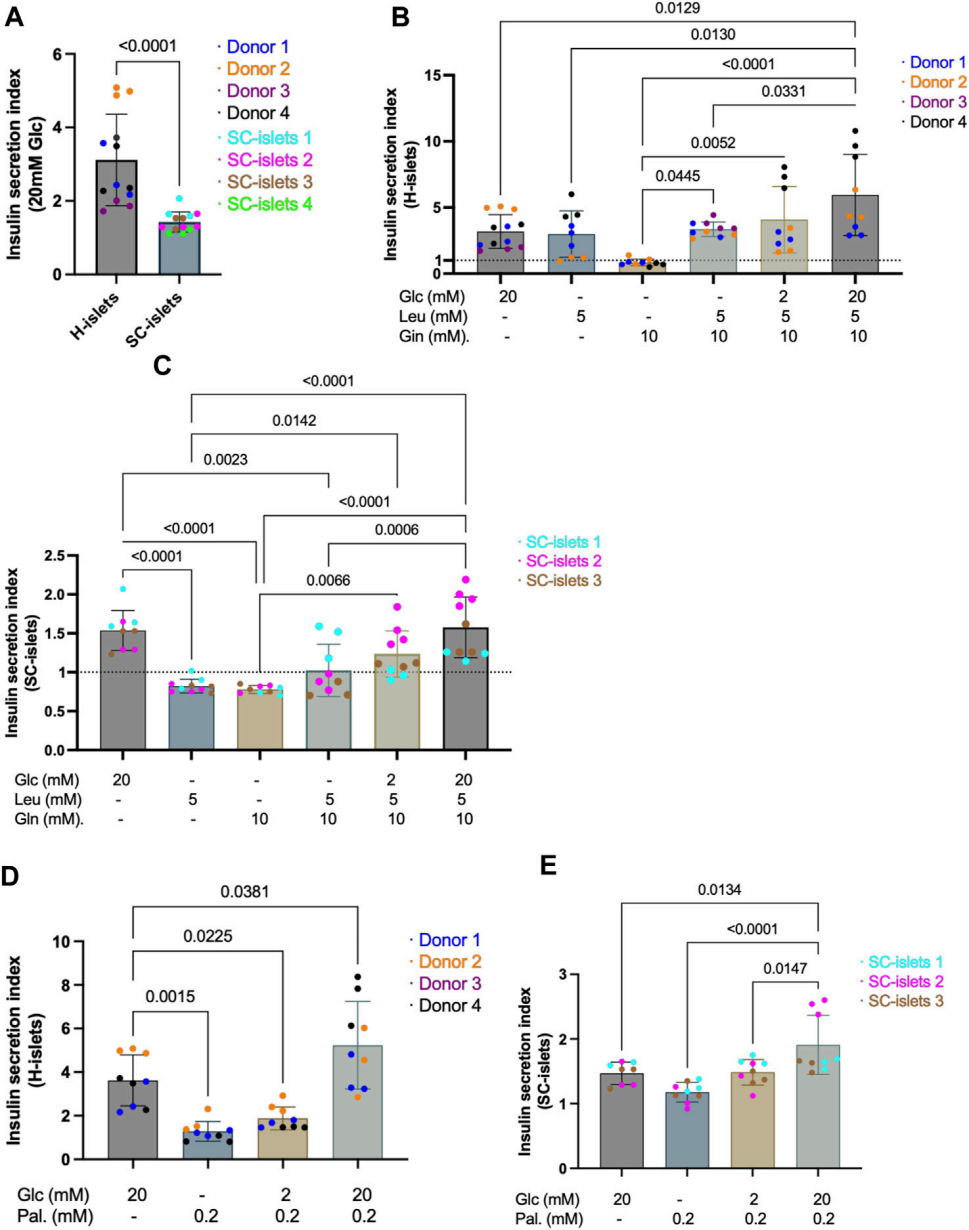
SC-islets and human islets’ response to different nutrients. **(A)** SI of human islets and SC-islets in response to 20 mM Glc (glucose). Human islets: n = 4 donors, N = 3-4, SC-islets: *n* = 4, N = 3–4. Statistic: Mann-Whitney test. **(B)** SI of human islets in response to Glc, Leu (leucine), and Gln (L-glutamine) in different combinations and concentrations. The dotted line represents SI = 1. *n* = 3-4, N = 3–4. Statistic: one-way Anova with Turkey’s multiple comparison. **(C)** SI of SC-islets in response to Glc, Leu, and Gln in different combinations and concentrations. The dotted line represents SI = 1. *n* = 3, N = 3–4. Statistic: one-way Anova with Turkey’s multiple comparison. **(D)** Insulin SI of human islets in response to Glc and Pal (palmitate) separately and in combination. *n* = 3-4, N = 3–4. Statistic: one-way Anova with Turkey’s multiple comparison. **(E)** Insulin SI of SC-islets in response to Glc and Pal separately and in combination. *n* = 3, N = 3. Statistic: one-way Anova with Turkey’s multiple comparison. All data are plotted as mean ± SD.

To assess whether the information collected with SC-islets as disease models is translatable to that in human islets, we investigated the insulin secretion of SC-islets (during weeks 2-3 of stage 6) in response to different nutrient sources and benchmarked to human islets. Human primary islets showed a glucose SI of 3.13, significantly higher compared to 1.43 observed from 4 independent batches of SC-islets (Figure 4A, Supplementary Figure S5A, A’). Next, adding 5 mM of leucine alone in Krb without glucose strongly stimulates insulin secretion in human islets, with SI = 2.99, whereas SC-islets were unresponsive (SI = 0.83) (Figures 4B,C, Supplementary Figure S5B, B’). Glutamine is an important energy substrate for many body tissues, and is the most abundant amino acid in the blood, with a normal circulation concentration maintained at 0.6–0.9 mM (Henquin et al., 2006). A 10 mM of glutamine alone in Krb without glucose could not induce insulin secretion neither in human islets (SI = 0.85) nor in SC-islets (SI = 0.78) (Figures 4B,C, Supplementary Figure S5C, C’). The SI in response to a mix of 5 mM of leucine and 10 mM of glutamine without glucose in Krb was 4.71 for human islets, whereas the SI was 1.02 for SC-islets (Figures 4B,C, Supplementary Figure S5D, D’). The glutamine does not significantly increase the glucose stimulated insulin secretion index neither for human islets nor SC-islets, which showed that glutamine is not a primary insulin secretagogue in the categories of small amino acids. Interestingly, leucine stimulated insulin secretion in human islets but not in SC-islets. However, the mixture of 2 mM glucose, 5 mM leucine and 10 mM glutamine increased the SI for SC-islets from unresponsive to 1.20, whereas the SI for human islets was 4.08 (Figures 4B,C, Supplementary Figure S5E, E’). When 20 mM glucose presented together with a leucine-glutamine mixture, the SI for human islets increased to 5.96, whereas the SI for SC-islets increased to 1.53 (Figures 4B,C, Supplementary Figure S5F, F’). It has been reported that low-protein content diets induce mild insulin secretion, whereas a high-protein content diet meal potentiates the insulinemic response (Regazzi et al., 2016). Overall, our data suggested that leucine could stimulate insulin secretion in human islets but not in SC-islets. In addition, 5 mM leucine plus 20 mM glucose showed a trend of potentiated insulin secretion in human islets but not in SC-islets (Figures 4B,C).

Palmitic acid is the most common saturated fatty acid in the human body (Carta et al., 2017). To investigate the ability of SC-islets to secrete insulin in response to fatty acid and fructose, we incubated the cells in 0.2 mM palmitate together with different concentrations of glucose. The SI for human islets incubated with 0.2 mM palmitate alone was 1.28, whereas the SI for SC-islets was 1.18 (Figures 4D,E, Supplementary Figure S5G, G’). When 2 mM glucose presents together with 0.2 mM palmitate, the SI for human islets was 1.88 and for SC-islets increased to 1.49 (Figures 4D,E, Supplementary Figure S4H, H’). When 20 mM glucose presents together with 0.2 mM palmitate, the SI for human islets increased to 5.23, and the SI for SC-islets increased to 1.91 (Figures 4D,E, Supplementary Figure S5I, I’). The fructose was added with different concentrations together with 2 mM glucose. 2-, 5-, 10-, and 20-mM fructose could not induce significant insulin secretion in both human and SC-islets (Supplementary Figure S5J). In summary, palmitate alone could not stimulate insulin secretion in human islets and SC-islets. However, when 0.2 mM palmitate was added to 20 mM glucose in Krb, the SI was significantly higher than 20 mM glucose alone in stimulating insulin secretion in SC-islets (Figures 4D,E). Thus, our data revealed that palmitate could potentiate insulin secretion of SC-islets, consistent with previous observations in human islets that the presence of palmitate plus glucose increases insulin secretion than glucose alone (Parker et al., 2003; Cen et al., 2016; Kristinsson et al., 2017; Kuok et al., 2019).

### Intermediate metabolites stimulation revealed defective glycolysis in SC-islets

Although steady improvements have been made, the current SC-islets differentiation protocols are often unable to achieve an equivalent function to that of human primary islets (Davis et al., 2020). We observed a significantly lower GSIS index of SC-islets from our preparations and the different responses to nutrient sources compared to human islets (Figure 4). It has been evidenced that reduced anaplerotic cycling in the mitochondria causes reduced GSIS in SC-islets (Davis et al., 2020). To investigate the metabolism chain sufficiency of our SC-islets, we checked insulin secretion response with several inter-metabolites and benchmarked to human islets.

**FIGURE 5:**
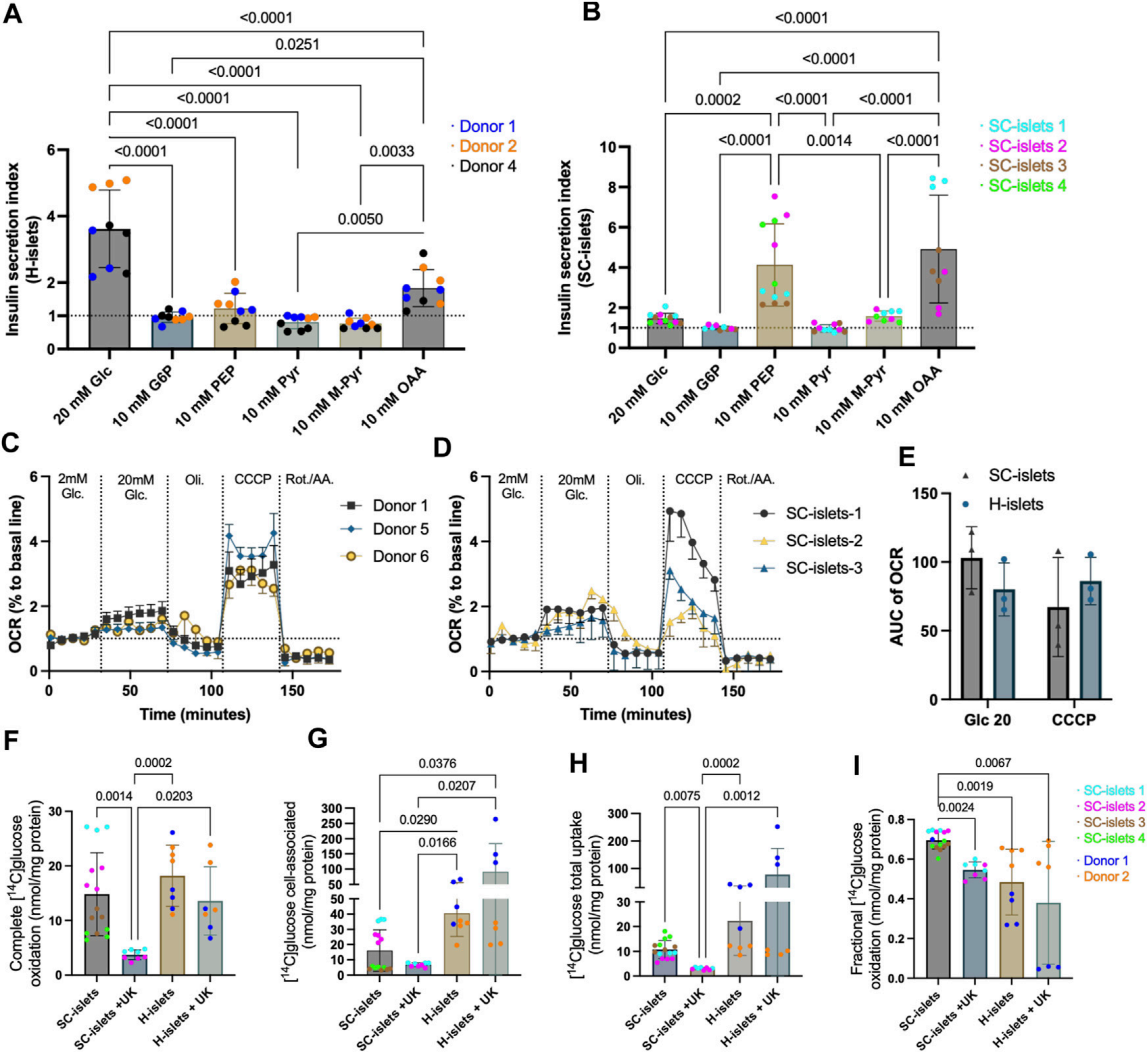
SC-islets and human islets’ response to different inter-metabolites and glucose oxidative status. **(A)** Insulin SI of human islets (H-islets) and SC-islets in response to Glc (Glucose), G6P (Glucose 6-phosphate), PEP (phosphoenolpyruvic), Pyr (pyruvate), M-Pyr (Methyl-pyruvate), and OAA (oxaloacetate acid). The dotted line represents SI = 1. Statistical test: One-way ANOVA with Turkey’s multiple comparisons test. *n* = 3, N = 3. **(B)** Insulin SI of SC-islets in response to Glc, G6P, PEP, Pyr, M-Pyr, and OAA. The dotted line represents SI = 1, results are displayed as mean ± SD. Statistical test: One-way ANOVA with Turkey’s multiple comparisons test. *n* = 3-4, N = 3–4. **(C, D)** Oxygen consumption rate (OCR) measurement for human islets **(C)** and SC-islets **(D)**. The dotted line represents 100% to basale line. *n* = 3, N = 4; the data was normalized to the mean of the basal OCR of each technical replicate. Results are displayed as mean ± SEM. **(E)** AUC analysis of OCR for human islets and SC-islets. Statistical test: Two-way ANOVA with Turkey’s multiple comparisons test. *n* = 3 **(F–I)** Substrate oxidation assay with D-[^14^C] glucose for human islets and SC-islets. Statistical test: Kruskal-Wallis test with Dunn’s multiple comparisons test. For human islets: *n* = 2, N = 4; for SC-islets: *n* = 2 -4, N = 4.

Glucose 6-phosphate (G6P) is the first intermediate of glucose metabolism, it bears a negative charge and cannot cross the cell membrane (Rajas et al., 2019). The SI for human islets incubated with 10 mM glucose 6-phosphate (G6P) alone was 0.96, whereas the SI for SC-islets was 1.00 (Figures 5A,B, Supplementary Figure S6A, A’). Phosphoenolpyruvate (PEP) is converted into ATP and pyruvate by pyruvate kinase (PK), which underlies β-cell sensing of both glycolytic and mitochondrial fuels (Lewandowski et al., 2020). The SI for human islets incubated with 10 mM PEP alone was 1.22, whereas the PEP significantly increased the insulin secretion for SC-islets compared to glucose SI (SI = 4.13) (Figures 5A,B, Supplementary Figure S6B, B’). It has been reported that SC-islets can actively respond to pyruvate stimulation in a dynamic profusion condition, while human islets are less responsive to pyruvate. This phenomenon has been characterized as an “immaturity” of SC-islets (Balboa et al., 2022). We could not observe a significant response to pyruvate in terms of insulin secretion from both human islets (SI = 0.80) and SC-islets (SI = 0.98) under a static incubation condition (Figure 5C, Supplementary Figure S6C, C’). Under a dynamic profusion condition, 10 mM pyruvate could not induce insulin secretion in both human islets and SC-islets, and the following 20 mM glucose perfusion massively induced insulin secretion in human islets but not in SC-islets (Supplementary Figure S6E, E’).

Unlike pyruvate, which barely triggers insulin secretion, methyl pyruvate (M-Pyr) has been proposed as a potent mitochondrial substrate and is widely used to study beta cell stimulus-secretion coupling (Düfer et al., 2002; Düfer et al., 2002). It has been reported that M-Pyr depolarize beta cells in a concentration-dependent manner from 5 to 20 mM, and stimulates insulin secretion at 20 mM (Zawalich and Zawalich, 1997; Lembert et al., 2001). To keep the equimolar mass as other insulin secretagogues used for this study, we incubated human islets and SC-islets with 10 mM M-Pyr, and observed an increase of insulin secretion in SC-islets with an SI = 1.58, however, no statistic significance when compared to cell-impermeable G6P stimulation (Figure 5B). In addition, 10 mM M-Pyr could not significantly induce insulin secretion in human islets (SI = 0.77) (Figure 5D, Supplementary Figure S6D, D’). Interestingly, 20 mM of M-Pyr induced insulin secretion under a dynamic profusion condition (Supplementary Figure S6F, F’). Almost half of pyruvate is converted to oxaloacetic acid (OAA) (anaplerosis) inside mitochondria in beta cells (Prentki et al., 2013). 10 mM of OAA slightly stimulated insulin secretion for human islets (SI = 1.83), whereas had a higher magnitude effect for SC-islets (SI = 4.9) (Figures 5A,B, Supplementary Figure S6G, G’).

Glucose-induced mitochondrial respiration ability is highly correlated with GSIS ability (Davis et al., 2020). To investigate the glucose metabolism chain, we analyzed the real-time oxygen consumption rate (OCR) during glucose stimulation and different mitochondrial respiration modulators, including Oligomycin, Carbonyl cyanide 3-chlorophenylhydrazone (CCCP), and Antimycin A & Rotenone. We observed that 20 mM of glucose increased mitochondrial respiration in human islets (Figure 5C) and SC-islets (Figure 5D). The area under the curve (AUC) analysis indicated that the OCR of human islets and SC-islets under glucose stimulation has no significant difference (Figure 5E).

Glucose sensing, followed by uptake and oxidative metabolism, is tightly regulated by the beta cells in islets to maintain glucose homeostasis in the body (Davis et al., 2020). To investigate the glucose uptake and oxidative metabolism in SC-islets, we traced glucose metabolism by 0.2 mM D-[^14^C] glucose with our previously developed method (Supplementary Figure S6H) (Wensaas et al., 2007). The completed glucose oxidation measured by the trapped ^14^CO_2_ was similar in SC-islets to that in human islets (Figure 5F). In addition, once a mitochondrial pyruvate carrier (MPC) blocker, UK-5099, was added to the incubation buffer, the glucose oxidation was significantly inhibited in SC-islets, whereas only a decreasing trend observed in human islets (Figure 5F), indicating that the ^14^CO_2_ production was highly dependent on following mitochondrial metabolism after glycolysis in SC-islets and human islets.

Cell-associated D-[^14^C] glucose measurement revealed a lower glucose accumulation in SC-islets than in human islets (Figure 5G). The MPC blocking induced a higher glucose accumulation that is three times higher in human islets than in SC-islets. In contrast, glucose accumulation induced by MPC blockers slightly decreased in SC-islets (Figure 5G). The total glucose uptake for SC-islets was similar to human islets, (Figure 5H), which is consistent with previous observation (Davis et al., 2020). However, MPC blocking increased the total glucose uptake in human islets and significantly decreased glucose uptake in SC-islets (Figure 5H). The glucose oxidation fraction (“completed glucose oxidation”/(“completed glucose oxidation” + “cell-associated glucose”)) was lowered by MPC blocking in both human islets and SC-islets (Figure 5I). Surprisingly, the glucose oxidation fraction was significantly higher in SC-islets than in human islets (Figure 5I). Previous data revealed insufficient glycolysis in SC-islets is a bottleneck that reduces insulin secretion compared with human islets (Davis et al., 2020). The pyruvate-mitochondrial transportation cutting off dismissed the glucose uptake and accumulation in SC-islets but not in human islets, supported that the glycolysis may not be sufficient even at a basal respiration condition (0.2 mM glucose).

### Insulin secretion of SC-islets with aberrant glucose metabolism trafficking improved by pyruvate kinase agonist TEPP46

**FIGURE 6:**
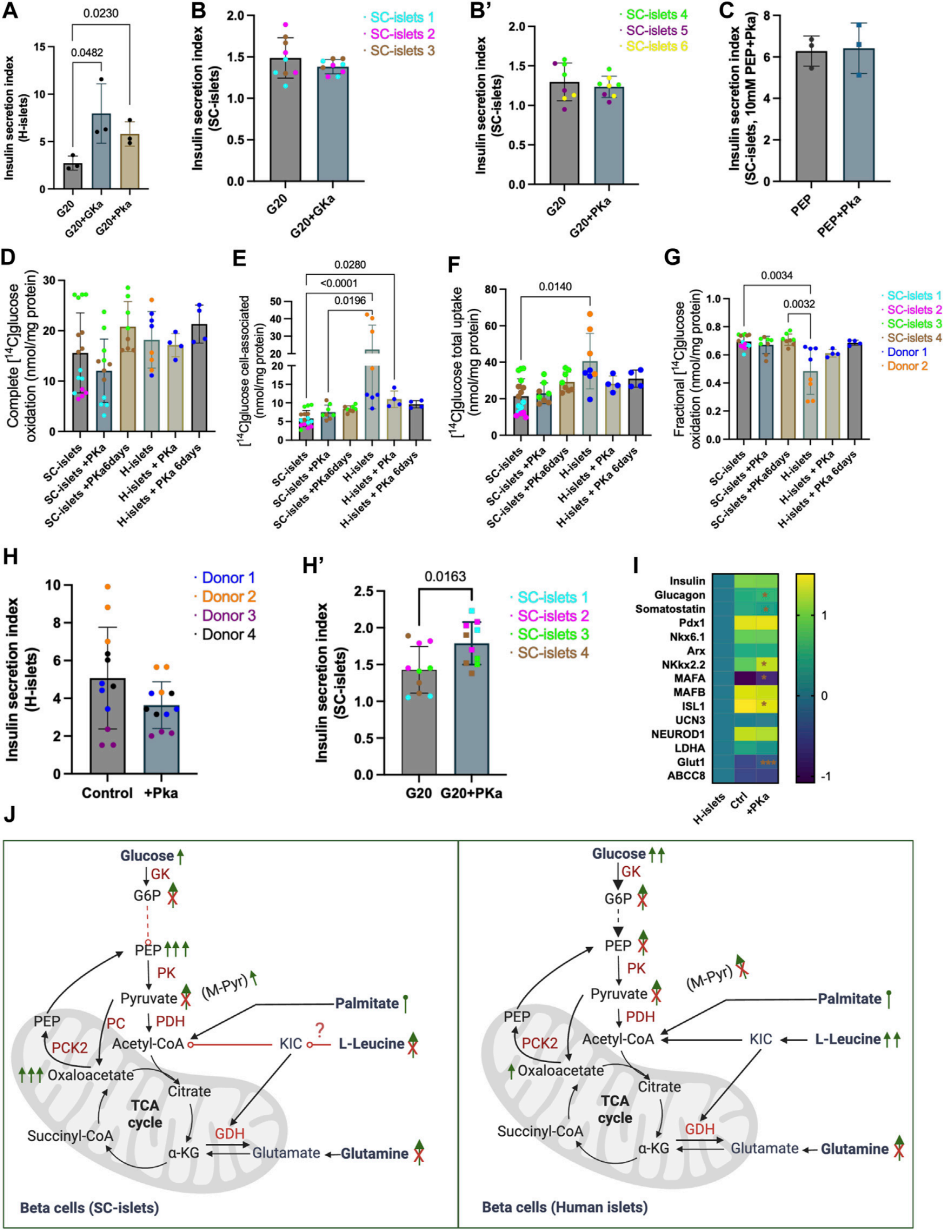
Pyruvate kinase agonist TEPP46 improves insulin secretion of SC-islets with aberrant glucose metabolism trafficking. **(A)** Human islets’ acute response to 0.01 mM PKa or 0.1 mM GKa mixed with 20 mM Glc. Statistical test: Unpaired *t*-test. N = 3 **(A)** second donor’s reactions are shown in Supplementary Figure S7A). **(B)** SC-islets’ acute response to 0.1 mM GKa mixed with 20 mM Glc. Statistical test: unpaired *t*-test with Welch’s correction. *n* = 3, N = 3. **(B′)** SC-islets’ acute response to 0.01 mM PKa mixed with 20 mM Glc. Statistical test: unpaired *t*-test with Welch’s correction. *n* = 3, N = 2–3. **(C)** SC-islets’ acute response to 10 mM PEP mixed with 0.01 mM PKa. Statistical test: Unpaired *t*-test. N = 3 technical replicates. **(D–G)** Substrate oxidation assay with D-[^14^C] glucose for human islets and SC-islets. Statistical test: Kruskal-Wallis test with Dunn’s multiple comparisons test. For human islets: *n* = 1-2, N = 4, for SC-islets: *n* = 2-3, N = 4. Donor 2 and SC-islets 2 are only present in the untreated groups. **(H)** Insulin SI for human islets after 6 days incubation with 0.01 mM PKa. Statistical test: Unpaired two-tailed *t*-test. *n* = 4, N = 3. **(H′)** Insulin SI for SC-islets after 6 days incubation with 0.01 mM PKa. Statistical test: Unpaired two-tailed *t*-test. *n* = 4, N = 2–3. **(I)** RT-qPCR analysis for SC-islets after 6 days incubation with 0.01 mM PKa. “H-islets”, human islets; “Ctrl”, SC-islets without TEPP46 treatment, “+Pka”, SC-islets with TEPP46 treatment for 6 days. Statistical test: Unpaired two-tailed *t*-test. *n* = 3 human islets, N = 2; *n* = 3 independent wells for SC- islets, N = 2. *p < 0.05, ***p < 0.001. **(J)** Schematic depicting the SC-islets and human islets’ response to different nutrients and inter-metabolites. List of abbreviation see Supplementary Table S5.

To investigate how the SC-islets repones to potential antidiabetic drugs, we tested a classical glucokinase activator, MK-0941 (Meininger et al., 2011), and a recently developed pyruvate kinase activator (PKa), TEPP46 (Lewandowski et al., 2020) in SC-islets. Glucokinase (GK) is expressed in beta cells and sets the metabolic flux rate to enable an instant response to blood glucose changes (Lewandowski et al., 2020). Thus, glucokinase activation (GKa) was initially predicted can enhance insulin section (Ashcroft et al., 2023). Pyruvate kinase (PK) converts ADP and PEP into ATP and pyruvate. Recent preclinical data showed that PK activation improved beta-cell function *in vitro* and *in vivo* (Abulizi et al., 2020; Corkey, 2020). Consistent with previous reports (Lewandowski et al., 2020), we observed an acute response to PKa and GKa in human islets regarding increased insulin secretion under glucose stimulation (Figure 6A). However, GK and PK activation could not significantly increase insulin secretion for SC-islets (Figure 6B, B’, Supplementary Figure S7D and E). By incubating the SC-islets with PEP and PKa to investigate if the PK activation can increase insulin secretion by bypassing the early glycolysis clog, we still cannot observe a significant change in SI (Figure 6C and Supplementary Figure S7F). The D-[^14^C] glucose oxidative analysis showed that PKa did not impact SC-islets’ oxidative status when PKa was added into the Krb buffer (Figure 6D).

Animal models and T2D patients with GKa treatments have been reported hypoglycaemic (Meininger et al., 2011), whereas PK activation improved whole-body metabolic health in diabetic rat (Abulizi et al., 2020), which demonstrated that PK activation by TEPP46 could be a potentially better antidiabetic drug. Thus, we incubated the human islets and SC-islets (during week 2–3 of stage 6) with PKa for 6 days to investigate the long-term effects. 6 days of incubation with PKa did not significantly alert SC-islets glucose oxidation (Figure 6D), accumulation (Figure 6E), uptake (Figure 6F), and the fraction of oxidized glucose status (Figure 6G), although upward trends were observable (Figures 6D,E). 6 days of incubation PKa did not increase the insulin secretion index for human islets (Figure 6H, Supplementary Figure S7G). Unlike the actuate incubation with PKa, the insulin secretion indexes were significantly increased in SC-islets after 6 days incubation with Pka (Figure 6H’, Supplementary Figure S7H). The mRNA expression analysis revealed that the beta cell maturation makers, such as *NKX2.2, MAFA,* and *Glut1* were significantly upregulated after the 6-day incubation with PKa (Figure 6I). It was not surprising that PKa failed to increase insulin secretion in SC-islets in acute situations as PEP can successfully elevate insulin secretion, which means the PK activity may already be sufficient in SC-islets (Figure 6J). Nonetheless, the long-term incubation with PKa significantly increased SI in SC-islets, indicating that the PK activation may have a different regulation mechanism for SC-islets other than directly modulating PK activity.

## Discussions

Diabetes research has faced persistent challenges due to the absence of genuine disease models that should ideally be unlimited and capable of replicating the abnormalities associated with the development, structure, and function of human islets in pathological conditions (Zhang et al., 2022). An appealing alternative for human islets for diabetes research, yet a significant challenge in the field involves creating mature and functional islet cells derived from stem cells, which has been increasingly explored recently (Rogal et al., 2019; Balboa et al., 2021; Goswami et al., 2022; Yin et al., 2022; Vandana et al., 2023). Despite enormous progress that has been made in generating beta cells from stem cells, the beta cell differentiation efficiency varies across different cell lines and labs, and functional maturation is not complete *in vitro* (Balboa et al., 2022; Fantuzzi et al., 2022; Iworima et al., 2024). To improve a lab-scale differentiation protocol that generates SC-islets for disease modeling and provide insights into insulin secretagogues when employing SC-islets for disease modeling, this study described an optimized protocol with increased pancreatic endocrine cell differentiation efficiency by integrating a WNT signaling inhibition following the DE stage compared to the untreated group. A tri-hormonal cell population (Cp+/GCG+/SST+) and its cell identity dynamics was defined within SC-islets. The SC-islets maintained their function for up to 8 weeks *in vitro,* thus providing a flexible time window for downstream applications. Moreover, SC-islets manifested diverse reactivity patterns when exposed to distinct nutrient sources and exhibited deviant glycolytic metabolic characteristics compared to human primary islets.

Cells that express multiple endocrine hormones concurrently, such as insulin and glucagon, are recognized as an immature cell type in the developing pancreas (Tremmel et al., 2023). Cp+/GCG + cells, namely bi-hormonal cells, were frequently reported in previous studies (Hogrebe et al., 2020; Mahaddalkar et al., 2020; Balboa et al., 2022; Tremmel et al., 2023), including in single-cell sequencing analysis among the final products of SC-islets differentiation (Shiraki and Kume, 2020; Wiedenmann et al., 2021; van Gurp et al., 2022; de la O et al., 2023). Somatostatin inhibits the secretion of both insulin and glucagon from the pancreas (Carril Pardo et al., 2022). The Cp+/SST+ and GCG+/SST + bi-hormonal cells were reported in zebrafish models of diabetes (Carril Pardo et al., 2022; Singh et al., 2022), lineage tracing experiments in mice (Gribben et al., 2021; Perez-Frances et al., 2022), and during beta cell differentiation from human stem cells (Leavens et al., 2021). A recent study reported that INS+/GCG + bi-hormonal cells significantly decreased while accomplished with an increase of GCG + cells and a decreased INS + cells from weeks 0–6 of stage 7 differentiation *in vitro* (Balboa et al., 2022). We observed similar bi-hormonal cell identity dynamics of Cp+/GCG + cells. However, these changes did not rely on peripheral small molecule inhibitors or growth factors in our observation. Moreover, the Cp+/GCG+/SST + cells, here named as tri-hormonal cells was reported in a study during stem cell differentiation, but its cell identity dynamics was not characterized (Nostro et al., 2011). We found that the tri-hormonal cells shared −10% of the SC-islets cells at week 2 of stage 6, and decreased to less than 1% at week 8 of stage 6. It was reported that Cp + cells in SC-islets have a less defined chromatin state than in human islets, and expresses a gradient of several hormonal genes other than a fixed cell identity *in vitro* (Augsornworawat et al., 2023). However, a recent abstract revealed a prevalence of tri-hormonal islet cells (0.45%) and bi-hormonal islet cells are present within human pancreatic islets throughout life, but the detailed information awaits the full manuscript to be published (HAHM et al., 2023). The increase in Cp+/GCG + bi-hormonal cells under diabetic conditions in humans and mice has been reported (Katahira et al., 2020). In contrast, conflicting results regarding islet cell plasticity have also been reported, such as the beta cell mass could increase, but cell identity remained unchanged in *in vivo* obesity models (Cox et al., 2016; Yamamoto et al., 2017). Thus, the increase of single hormonal cell commitment observed in this study under prolonged culturing *in vitro* cannot be interpreted as an increase in cell maturity. Nonetheless, our data supported that the current EFSM media without small molecule inhibitors or growth factors can maintain the SC-islets’ function for up to 8 weeks *in vitro*, which provides additional flexibility for downstream applications.

Nutrient control of insulin secretion has been well studied in human islets (Düfer et al., 2002; Henquin et al., 2006; Patterson et al., 2014; Regazzi et al., 2016). This study investigated the role of different nutrient sources, such as glucose, amino acids, and palmitate, in stimulating insulin secretion in SC-islets. It was unexpected that the leucine and its combination with glutamine failed to trigger significant insulin secretion in SC-islets, similar to what observed in adult human and mouse islets (Helman et al., 2020), but differed from what was observed in human primary islets (this study) and others reported in SC-islets (Helman et al., 2020; Balboa et al., 2022). Leucine is a known amplifier of insulin secretion, yet there is ongoing debate about its mechanisms of action (Kolic et al., 2023). Therefore, the underlying mechanism of why leucine failed to trigger insulin secretion needs head-to-head comparison and further investigation. Chronic treatment with palmitate induces insulin hypersecretion and later impairment of human islet function and has frequently been used for obese and diabetes modeling *in vitro* (Cen et al., 2016; Kristinsson et al., 2017; Groebe et al., 2018). Henquin et al. observed acute insulin stimulation by palmitate in human islets in 2006 (Henquin et al., 2006). However, to the best of our knowledge, the acute effects of palmitate in SC-islets are missing. Our data revealed that palmitate amplifies insulin section in both human primary islets and SC-islets. To this end, the SC-islets would be suitable for obese modeling, and our previous work has evaluated palmitate’s long-term effects on SC-islets (Aizenshtadt et al., 2024).

Intermediate metabolites stimulation data supports previous investigation that the glycolysis flux is insufficient in SC-islets compared to that in human islets (Davis et al., 2020; Barsby et al., 2023). Although the SC-islets showed a higher complete oxidation with ^14^C-glucose metabolism analysis, the cellular glucose accumulation in SC-islets under MPC blocking was negligible compared to that in human islets, which may indicate the glucose transport was not as sufficient as in human primary islets. The acute insulin secretion induced by GKa and PKa in SC-islets was negligible, which may be due to the aberrant metabolite trafficking in SC-islets, consistent with previous reports (Davis et al., 2020; Barsby et al., 2023). Interestingly, a 6-day incubation with PKa significantly improved the insulin secretion in SC-islets, although they showed an aberrant glycolysis ability. This may be because of increased markers of beta cell health and maturity markers’ expression, such as *MAFA* and *ISL1*, as reported in this study and previously observed in human islets (Abulizi et al., 2020). Furthermore, it has reported that the lack of functionality observed in SC-islets may be attributed to the insufficient expression of *GLUT1* in cells expressing insulin (Bruin et al., 2014). *GLUT1* was significantly upregulated after 6 days of incubation with PKa, further indicated that the TEPP46 has a broader impact on SC-islets than PK activation alone.

Limitations of this study include the validation of the protocol, which was only carried out with one ESC line. Although cell line H1 is widely used in SC-islets research, validating the protocol with multiple cell lines could be beneficial. Secondly, The considerable variability of human islet preparations from different donors makes them a problematic gold-standard to rely upon (Balboa et al., 2021; Fantuzzi et al., 2022). We found significant data deviations in human islets, which could be improved by better access to clinical-grade human islets for research. Thirdly, the pre-clinical stage anti-diabetic drug, TEPP46, applied in this study as an initial example of drug evaluation, was lacking in dose and enzyme activity determination. A project is ongoing to systematically evaluate the impact of this drug in a stem cell-derived multi-organ obese model.

In summary, this study validated and improved a planar SC-islets differentiation protocol, thus providing a low-cost research-scale SC-islets manufacturing option. The SC-islets recapitulated the main features of human primary islets, such as the cell components, ability to prevent alloxan-induced diabetes in mice, similar palmitate responses, and sufficient mitochondrial metabolism ability. The acute impact of different insulin secretagogues was carried out to provide insights for media design when employing SC-islets for disease modeling, especially when multiple organ systems wanted to be coupled (Figure 6G). This protocol and other protocols (Hogrebe et al., 2020; 2021) do not need to add external small chemicals or growth factors to maintain SC-islets function *in vitro,* provides convenience for downstream applications without worrying about the complex and maybe contradictive effects of different small chemicals used in SC-islets maintenance and drug screening. On the other hand, we should carefully choose the readouts in downstream applications due to several significant limitations of SC-islets, such as cell population dynamics under prolonged culturing *in vitro* and the defective glycolysis flux in SC-islets.

## Materials and methods

### Cell culture and differentiation

SC-islets were generated from human embryonic stem cell line H1 (Coriell Institute for Medical Research). Undifferentiated H1 cells were cultured in Essential 8™ media (A1517001, ThermoFisher) on tissue culture plates coated with Geltrex™ (A1413201, ThermoFisher) in a humidified incubator containing 5% CO_2_ at 37°C. The H1 cell clones were passaged as clumps in every 2–4 days by 0.5 mM EDTA dissociation. To initiate SC-islets differentiation, H1 cells were counted with NucleoCounter NC-202 cell counter (ChemMetec) and seeded as single cells at 2 × 10^5^ cells/cm^2^ in 1:50 diluted Geltrex™ coated cell culture plates for 24 h. Cells were then washed with DPBS and incubated with stage 1 media supplemented with 3 μM CHIR99021 (S2924, Selleckchem) and 100 ng/mL Activin A (120–14, PeproTech) for day 1 of stage 1. On days 2–4, the cells were incubated with stage 1 media supplemented with 100 ng/mL Activin A. At stage 2, the cells were incubated for 2 days with stage 1 basal media supplemented with 0.25 mM vitamin C (A4544, Sigma), 1.25 µM IWP2 (3533, Bio-Techne) and 50 ng/mL KGF (AF-100–19, PeproTech). At stage 3, the cells were incubated for 2 days with stage 3 basal media supplemented with 0.2 µM LDN193189 (6053, Tocris), 0.2 µM TPPB (HY-12359, MedChemExpress), 2 µM Retinoid Acid (R2625, Sigma), 0.25 µM SANT1 (C23H27N5, Sigma) and 50 ng/mL KGF. At stage 4, the cells were incubated for 4 days with stage 3 basal media supplemented with 0.1 µM Retinoid Acid, 0.25 µM SANT1, 0.2 µM LDN193189, 0.2 µM TPPB and 50 ng/mL KGF. At stage 5, the cells were incubated with stage 5 basal media supplemented with 0.1 µM Retinoid Acid, 0.25 µM SANT1, 0.2 µM LDN193189, 10 µM ALK5 inhibitor II (ALX-270–445-M005, Enzo Life Sciences), 1 µM T3 (T6397, Sigma), 1 µM Xxi (565789, Sigma). 1 μM Latrunculin A (3973, Bio-Techne) was added during the first day of stage 5. At stage 6, the cells were cultured in enriched serum-free medium (ESFM) with media change every day for 7 days. On day 7 of stage 6, the cells were dissociated with TrypLE Express (12604013, Gibco) and 4 million cells in 4 mL ESEM media were seeded in each well of ultra-low attachment cell culture plate (CLS3261, Sigma). The cells were then aggregated and maintained as spheroids on an orbit-shaker (Thermo Fisher) at 100 RPM for over 7 days till analysis or drug treatment. The media was changed every other day after day 7 of stage 6. The basal media were as follows. Stage 1: MCDB 131 (10372–019, Invitrogen) supplemented with 1% P/S, 100x GlutaMAX (35050079, Invitrogen), 0.5% BSA (68700, Proliant), 1.174 g/L NaHCO_3_ (S6297, Sigma), 4.5 mM glucose (G7021, Sigma). Stage 3: MCDB 131 supplemented with 1% P/S, 100x GlutaMAX (35050079, Invitrogen), 2% BSA, 1.154 g/L NaHCO_3_, 4.5 mM glucose, and 0.25 mM vitamin C. Stage 5: MCDB 131 supplemented with 1% P/S, 1x GlutaMAX, 2% BSA, 1.154 g/L NaHCO_3_, 14.5 mM glucose, 0.25 mM vitamin C, 10 μg/mL Heparin (H3149, Sigma) and 200x ITS-X (51500056, ThermoFisher). ESFM: MCDB 131 supplemented with 1% P/S, 100x GlutaMAX, 100x NEAA (11140050, Gibco), 2% BSA, 1000x Trace Elements A (Corning, 25-021-CI), 1000x Trace Elements B (Corning, 25-022-CI), and 10 µM ZnSO4 (Z0251, Sigma). The WNT inhibition was conducted by adding 0.25 µM of IWP2 in the completed differentiation either at stages 2, 4 or 5.

### Human primary islets preparation

Human primary islets from non-diabetic brain-dead multiorgan donors were obtained from the Nordic Network for Islet Transplantation (Oslo University Hospital, Rikshospitalet) after appropriate informed consent from relatives for multi-organ donation and use in research, and approved by the Regional Ethics Committees (REK 67671 and REK 270665). Human islets were cultured in CMRL 1066 (15-110-CV, Corning) supplemented with 5% human AB serum (000921, Milan Analytica AG), 20 mM HEPES, and 1% P/S in 6 well ultra-low attachment plates. Islet donor characteristics are listed in Supplementary Table S1. The human islets were maintained in a humidified incubator containing 5% CO_2_ at 37°C for a maximum of 3 weeks and the analysis and drug treatment were conducted within these 3 weeks. The media were exchanged every two to 3 days.

### Immunofluorescence staining

SC-islets and human islets were harvested and fixed in 4% PFA for 30 min at 4°C. After blocking with blocking buffer (DPBS with 1% BSA, 0.1% Triton-X100) for 30 min at room temperature (RT), the spheroids were washed with DPBS with 1% BSA and placed in 30% sucrose (S0389, Sigma) at 4°C overnight. The spheroids were then embedded in O.C.T (23730571, Fisherscientific) and preserved at −80°C. The spheroids were cut into 10 µm thick slides with Cryostats (CM 1950, Leica). Cells during differentiation were fixed in 4% PFA for 15 min at RT, permeabilized and blocked with a blocking buffer for 30 min at RT. Animals were euthanized and grafts retrieved within 230 days of implantation. Mouse kidneys grafted with SC-islets were fixed in formalin and embedded in paraffin, and then cut 8 µm thick by Oslo university hospital. The paraffin was removed with Xylene (534056, sigma) and rehydrated with ethanol, and antigens retrieval was done with target retrieval solution (S236784-2, Dako) and boiling water bath. Primary antibodies were incubated overnight at 4°C, and secondary antibodies were incubated for 1 h at RT. Antibodies information used for immunofluorescence staining is listed in Supplementary Table S2. The images were taken with Zeiss LSM 700 confocal microscopy and Nikon Eclipse Ts2R-FL microscopy.

### Flow cytometry analysis

Cells during differentiation at each stage or the SC-islets were dissociated as single cells with TrypLE Express for 3 min at 37°C, and then fixed in 4% PFA for 15 min at RT, permeabilized and blocked with blocking buffer for 30 min at RT. Primary antibodies were incubated overnight at 4°C, and secondary antibodies were incubated for 1 h at RT. Cells after immunostaining were analyzed with LSR Fortessa II (BD Biosciences). Flow cytometry data were analyzed with FlowJo (Version 10.8.1). Over 50,000 cells were acquired for tSNE analysis (Maaten and Hinton, 2008). For cross sample comparism, the data set for each sample were then sampled with Downsample plugin (Version 3.3.1, Flowjo-exchange) to equally assign the same number of events for each sample (20,000 events after single cell gating), and then the three-channel fluorescence data were de-dimensioned with build in tSNE function with the Exact and FFT interpolation algorithm selected, and cell subpopulation was then clustered with FlowSOM plugin (Version 3.0.18) (Van Gassen et al., 2015).

### RNA extraction and RT-qPCR

Total RNA from the differentiated cells and human primary islets was isolated using TRIzol by following the manufacturer’s instructions. The cDNA was synthesized with a High-Capacity cDNA Reverse Transcription Kit (4368814, Applied Biosystems). PowerUp SYBR Green (A25780, Applied Biosystems) based real-time PCR was performed in Viia 7 real-time PCR system (Applied Biosystems) with the standard model. TBP (TATA box binding protein) was taken as an internal normalization control. Primers were provided in Supplementary Table S3.

### Transplantation studies

Animals were housed with no more than 5 mice per cage, under a 12 h light-dark cycle with free access to food and water except during fasting. 10-12-week-old male BALB/c Rag 1^−/−^ immunodeficient mice (C.129S7(B6)-*Rag1* ^*tm1Mom*^/J, stock 003145, The Jackson Laboratory) were transplanted with 600–700 SC-islets under the kidney capsule by using a 25G butterfly needle. The mouse’s blood glucose was monitored every week with blood glucose test strips (06453970170, Aviva). Approximately 60 µL of mice blood was taken in every 2 weeks for human C-peptide measurement with the human C-peptide ELISA kit (10–1136-01, Mercodia). At day 90 post transplantation 70 mg/kg alloxan (A7413, Sigma Aldrich) was administrated intravenously followed by monitoring of blood glucose weekly for the first 3 months of alloxan administration and every other week for the next 3 months. Mouse oral glucose tolerance tests (OGTT) were conducted 12 days after alloxan administration. Blood glucose was measured at 15, 30, 45, 60, and 120 min after glucose administration. The *in vivo* experiments were approved by the Norwegian National Animal Research Authority (FOTS ID 14263) and performed according to the guidelines for care and use of laboratory animals published by the US National Institutes of Health (NIH Publication, 8th Edition, 2011), and Norwegian Animal Welfare Act.

### Insulin secretion assays

30 SC-islets or 20 human primary islets were hand-picked into Transwell cell culture inserts (CLS3414, Sigma) placed in 24 well cell culture plates. The spheroids were washed three times and equilibrated in 1 mL Krebs-Ringer buffer (KRB: 128 mM NaCl, 5mM KCl, 1.2 mM MgSO_4_, 2.7 mM CaCl_2_, 1.2 mM KH_2_PO_4_, 1 mM Na_2_HPO_4_, 5 mM NaHCO_3_, 10 mM HEPES and 0.1% BSA) containing 2 mM glucose for 1 h at 37°C. The SC-islets or human islets were then incubated sequentially in KRB containing 2 mM glucose, KRB containing different nutrients or inter-metabolites, and KRB containing 2 mM glucose with 30 mM KCl for 60 min each. The supernatant after incubating in each step was harvested for detecting insulin production. Insulin was measured by human insulin ELISA kit (10-1113-10, Mercodia). Different chemicals used for insulin stimulation were listed in Supplementary Table S4.

### Oxygen consumption rate measurement

The oxygen consumption rate (OCR) analysis was performed under the Seahorse XFe24 analyzer (Agilent). 30 differentiated SC-islets or human islets were hand-picked and seeded in the XF24 24-well cell culture microplates, the spheroids were washed 3 times with RPMI-1640 media (R6504, Sigma) containing 0.1% BSA, and then equilibrated in assay media (RPMI-1640 media supplemented with 0.1% BSA and 2 mM glucose) for 2 h at 37°C in air. The basal respiration was determined in assay media and data was measured for 5 cycles with a 6-min interval. Followed by incubating the cells with 20 mM glucose to determine the OCR change upon glucose stimulation. The oxygen consumption at 20 mM glucose condition was measured for 6 cycles with a 6-min interval. The final concentration at 5 μM of oligomycin (9996L, Cell Signaling Technology), CCCP (Carbonyl cyanide 388 3-chlorophenylhydrazone, C2920, Sigma), Rotenone (R8875, Sigma) and Antimycin A (A8674, Sigma) were added sequentially. The oxygen consumption was then measured in 5 cycles with a 6-min interval. The OCR values were normalized to the averaged baseline values.

### Glucose metabolism: substrate oxidation assay

To examine glucose metabolism, human or SC-islets were differentiated as described previously and cultured in an ultra-low attachment treatment (ULA) Nunclon™ Sphera™ 96-well plate with D-[^14^C(U)]glucose (0.5 μCi/mL, 200 µM prepared in DPBS with BSA (15 μM) and HEPES (10 mmol/L)) (PerkinElmer NEN^®^, Boston, MA, United States) for 4 h following the previously described method (Supplementary Figure S6H) (Wensaas et al., 2007). Briefly, 1 M NaOH was used to activate a 96-well UniFilter^®^ microplate (PerkinElmer, Shelton, CT, United States) for capturing CO_2_ from metabolism and then placed on top of the 96-well plate during the incubation time. After 4 h, islets were then placed in small tubes (200 µL), washed with PBS, and harvested in 0.1 M NaOH. The ^14^CO_2_ trapped in the top filter (complete glucose oxidation), resulting from glycolysis and cellular respiration, and the cell-associated radioactivity were quantified by adding scintillation fluid (Ultima Gold XR, PerkinElmer) and counted using a 2450 MicroBeta2 scintillation counter (PerkinElmer). All results were adjusted for protein content, determined by Bio-Rad protein assay in VICTOR™ X4 Multilabel Plate Reader (PerkinElmer). The combined value of ^14^CO_2_ and the remaining cell-associated (CA) radioactivity were considered as an indicator of the total glucose uptake, represented as CO_2_ + CA. The fractional glucose oxidation was calculated as CO_2_/(CO_2_ +CA). Fractional oxidation represents the proportion of uptake that undergoes oxidation and may or may not correlate with complete oxidation.

### Statistical analysis

The “n” separates experiments from different biological replicates or independent stem cell differentiations or independent human donors; “N” separates experiments from technical replicates. Data is presented as mean ± standard deviation (SD) unless otherwise stated and GraphPad Prism software (10.2.0) was used for data analysis. The normal distribution of the data was assessed by the Shapiro-Wilks test before statistical analysis. If the data did not pass the normality test, the non-parametric option of the appropriate statistical test was used. Any outlier values in insulin secretion measurements were identified using ROUT with Q = 1% and excluded from statistical analysis and figure representation. Differences among the three groups were evaluated by one-way ANOVA or a two-way ANOVA followed by Dunnett’s or Turkey’s multiple comparison *post hoc* test. An unpaired *t*-test or a Mann-Whitney U test (for non-parametric) was performed for differences analysis between the two groups. Significance was set at *p* < 0.05.

## Data Availability

The original contributions presented in the study are included in the article/Supplementary Material, further inquiries can be directed to the corresponding authors.
