# Supplementary material for: Cell identity dynamics and insight into insulin secretagogues when employing stem cell-derived islets for disease modeling
Source: Front Bioeng Biotechnol. 2024 Jun 12;12:1392575. doi: 10.3389/fbioe.2024.1392575 (PMC11199790; doi:10.3389/fbioe.2024.1392575)
Supplement: Supplementary file 1 [file Presentation1.pdf]

Supplementary information:

Cell identity dynamics and insight into insulin secretagogues when employing stem cell-derived islets for disease modeling

Chencheng Wang[1,2*], Shadab Abadpour[1,2], Aleksandra Aizenshtadt[2], Andrea Dalmao Fernandez[6], Merete Høyem[1], Ingrid Wilhelmsen[2,5], Justyna Stokowiec[2], Petter Angell Olsen[2,5], Stefan Krauss[2,5], Simona Chera[3], Luiza Ghila[3], Helge Ræder[3,4], Hanne Scholz[1,2*].

[1]Department of Transplant Medicine and Institute for Surgical Research, Oslo University Hospital, Oslo, 0424, Norway,
[2]Hybrid Technology Hub, Center of Excellence, University of Oslo, Oslo, 0315, Norway,
[3]Department of Clinical Science, University of Bergen, Bergen, 5007, Norway,
[4]Department of Pediatrics, Haukeland University Hospital, Bergen, 5021, Norway.
[5]Department of Immunology and Transfusion Medicine, Oslo University Hospital, Oslo, 0424, Norway
[6]Section for Pharmacology and Pharmaceutical Biosciences, Department of Pharmacy, University of Oslo, Oslo, Norway
[*]Correspondence:
Chencheng.wang@medision.uio.no; hanne.scholz@medisin.uio.no

Supplementary Figures:

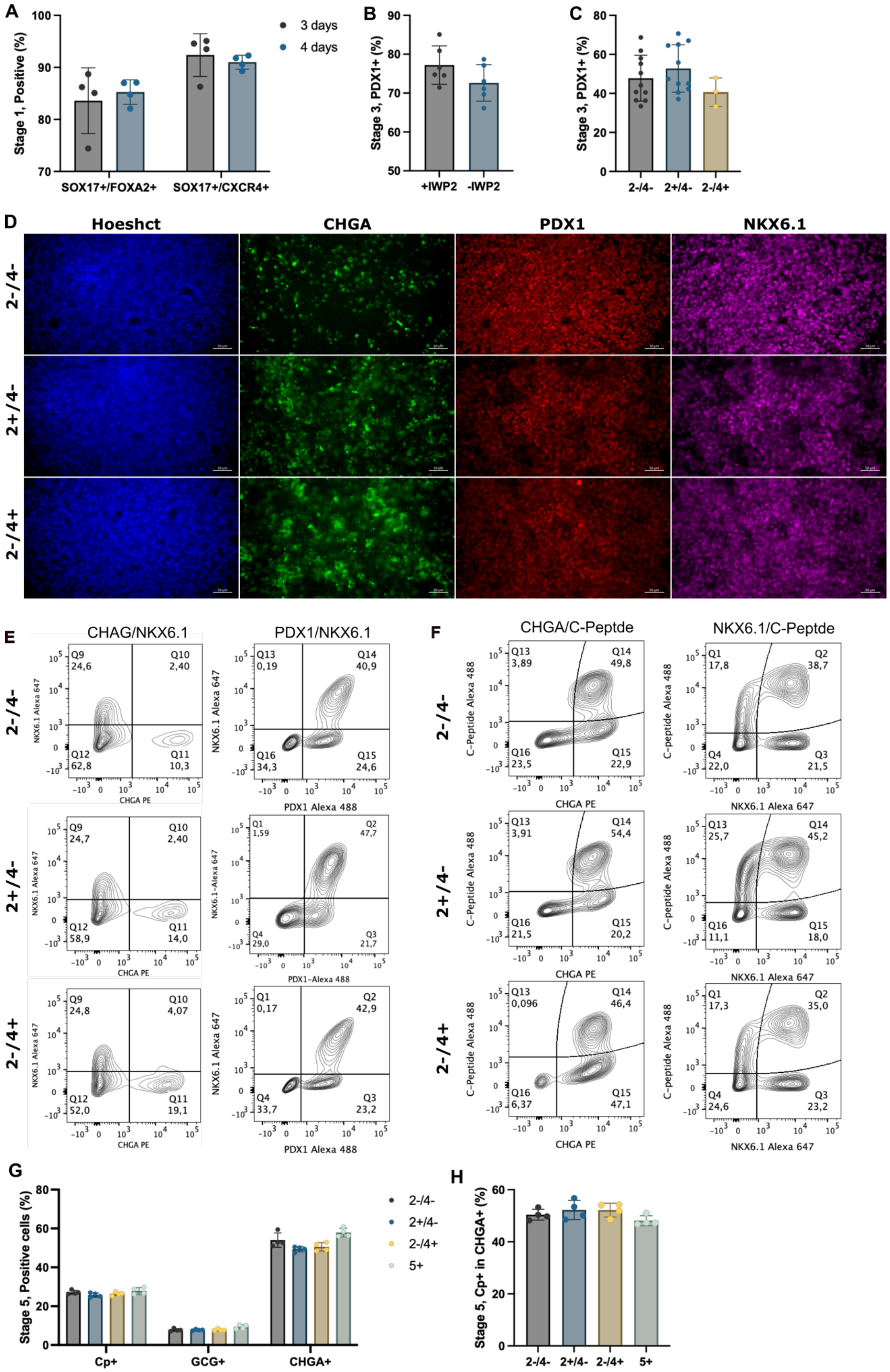

Figure S1. Evaluation during SC-islets differentiation.

(A) Flow cytometry quantification of DE cells at stage 1 with the marker SOX17, FOXA2, and CXCR4. Statistical test: Two-way ANOVA with Bonferroni's multiple comparisons test. n = 4. **(B)** Flow cytometry quantification of PDX1 positive cells at stage 3. n=6. **(C)** Flow cytometry quantification of PDX1+/NKX6.1+ cells at stage 4. Statistical test: Unpaired t test. n=3-11. **(D)** Representative immunostaining images for cells at stage 4 with or without IWP2 treatment. Arabic numeral representing the stages of differentiation; "+", with IWP2; "-" without IWP2. **(E)** Representative flow cytometry plots of stage 4 cells under different conditions for marker CHGA and NKX6.1. **(F)** Representative flow cytometry plots of stage 6 cells under different conditions for markers CHGA, NKX6.1 and C-peptide. **(G)** Flow cytometry quantification of stage 5 cells for Cp, GCG, and CHGA. Statistical test: Two-way ANOVA with Tukey's multiple comparisons test. n=4. **(H)** Flow cytometry quantification of Cp+ cells' percentage among CHGA+ cells at stage 5 under different conditions. Statistical test: Two-way ANOVA with Tukey's multiple comparisons test. n=4.

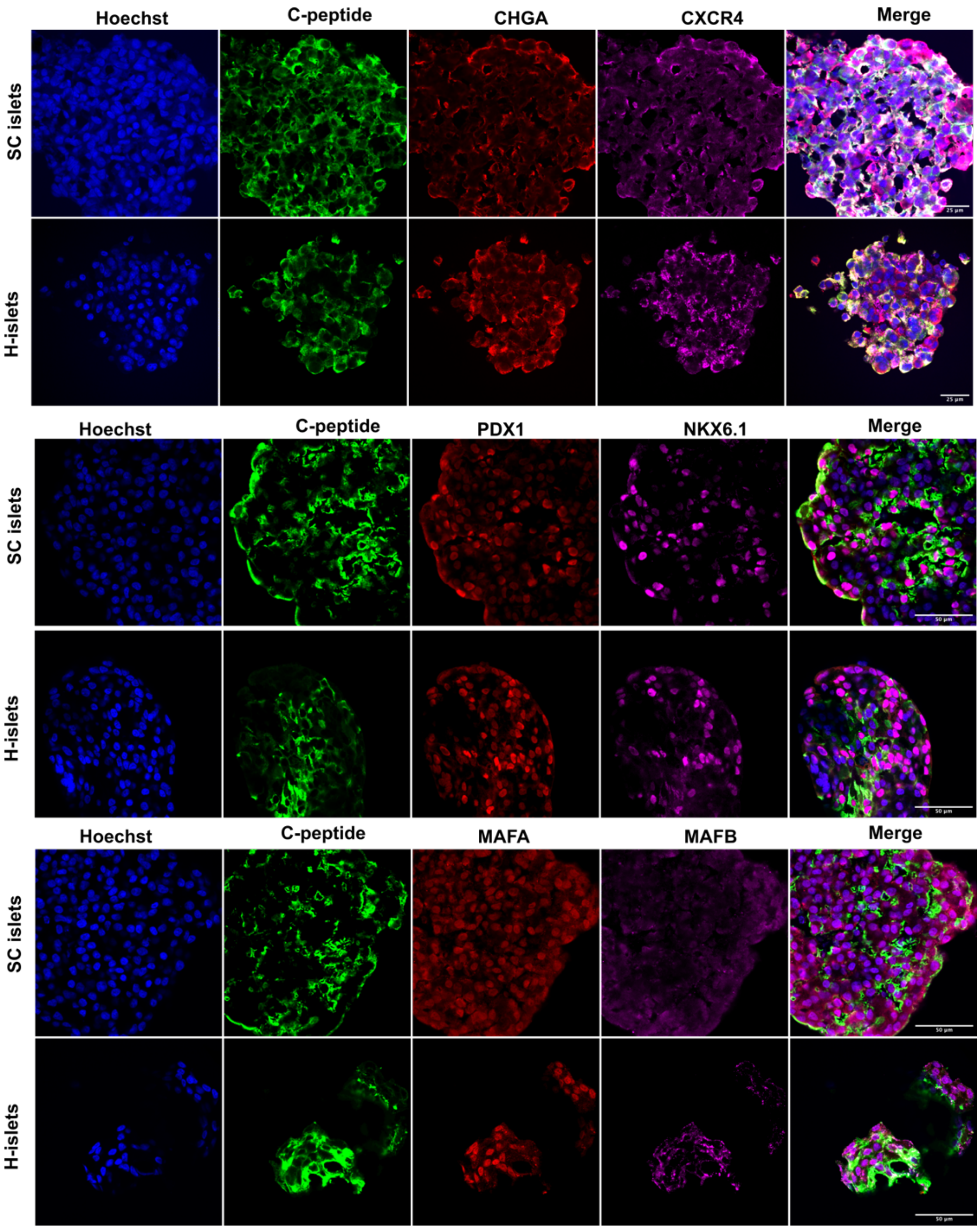

Figure S2. Immunostaining of SC-islets and human islets.
Representative immunostaining sections of SC-islets (week 2 of stage 6), and human islets (H-islets) for C-peptide, CHGA, CXCR4, PDX1, NKX6.1, MAFA, MAFB. Scale bar=50 µm; Nucleus stained with Hoechst 33342.

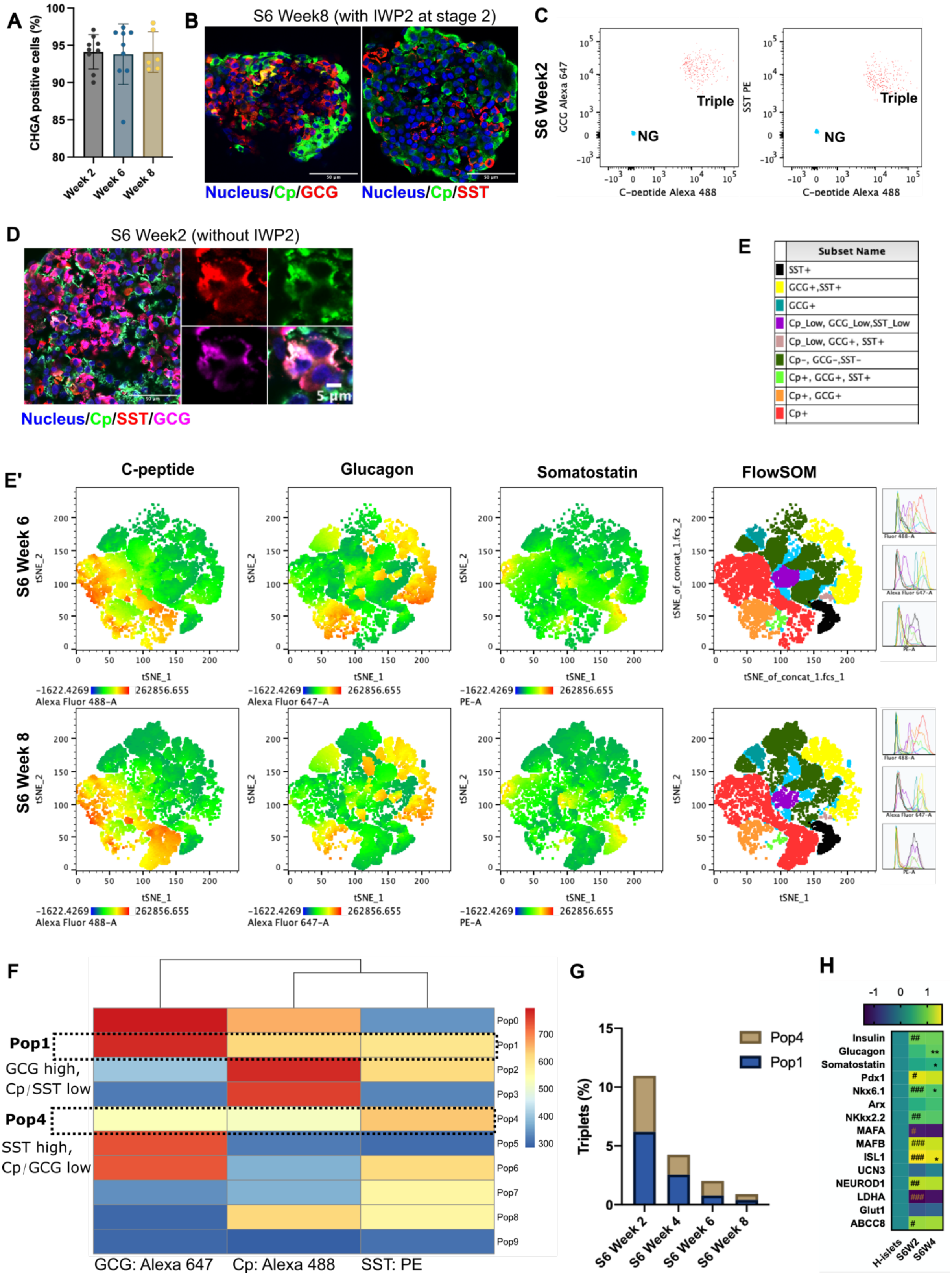

Figure S3. Evaluating SC-islets during prolonged culture *in vitro*.

(A) Flow cytometry quantification of SC-islets during prolonged culture in vitro for CHGA. Statistical test: Mann-Whitney test. n=6-9. **(B)** Representative immunostaining sections of SC-islets in week 8 of stage 6 for Cp, GCG, SST. Scale bar=50 µm; Nucleus stained with Hoechst 33342. **(C)** 2-dimensional flow cytometry plots of the Cp+/GCG+/SST+ subpopulation (triple) subtracted from tSNE analysis. "NG", Negative for Cp, GCG, and SST. **(D)** Representative immunostaining sections for SC-islets for Cp, SST, and GCG. SC-islets were generated without IWP2 treatment at stage 2. **(E)** Sub-cell populations that identified with FlowSOM were labeled Manually and annotated with different colors**. (E')** tSNE analysis of flow cytometry data for SC-islets under prolonged culturing in week 6 and 8. **(F)** FlowSOM analysis of flow cytometry data for SC-islets in week 2 of stage 6. **(G)** FlowSOM quantification of population (pop) 4 and 1 for SC-islets during prolonged culture in week 2/4/6/8. **(H)** RT-qPCR quantification of SC-islets in week 2 and week 4 of stage 6 and human islets. $^{*}p < 0.05$, $^{**}p < 0.01$, "*" represents the S6W4 Vs. S6W2 which determined by unpaired t-test. $^{\#}p < 0.05$, $^{\#\#}p < 0.01$, $^{\#\#\#}p < 0.001$, "#" represents the S6W2 Vs. H-islets which determined by unpaired t-test.

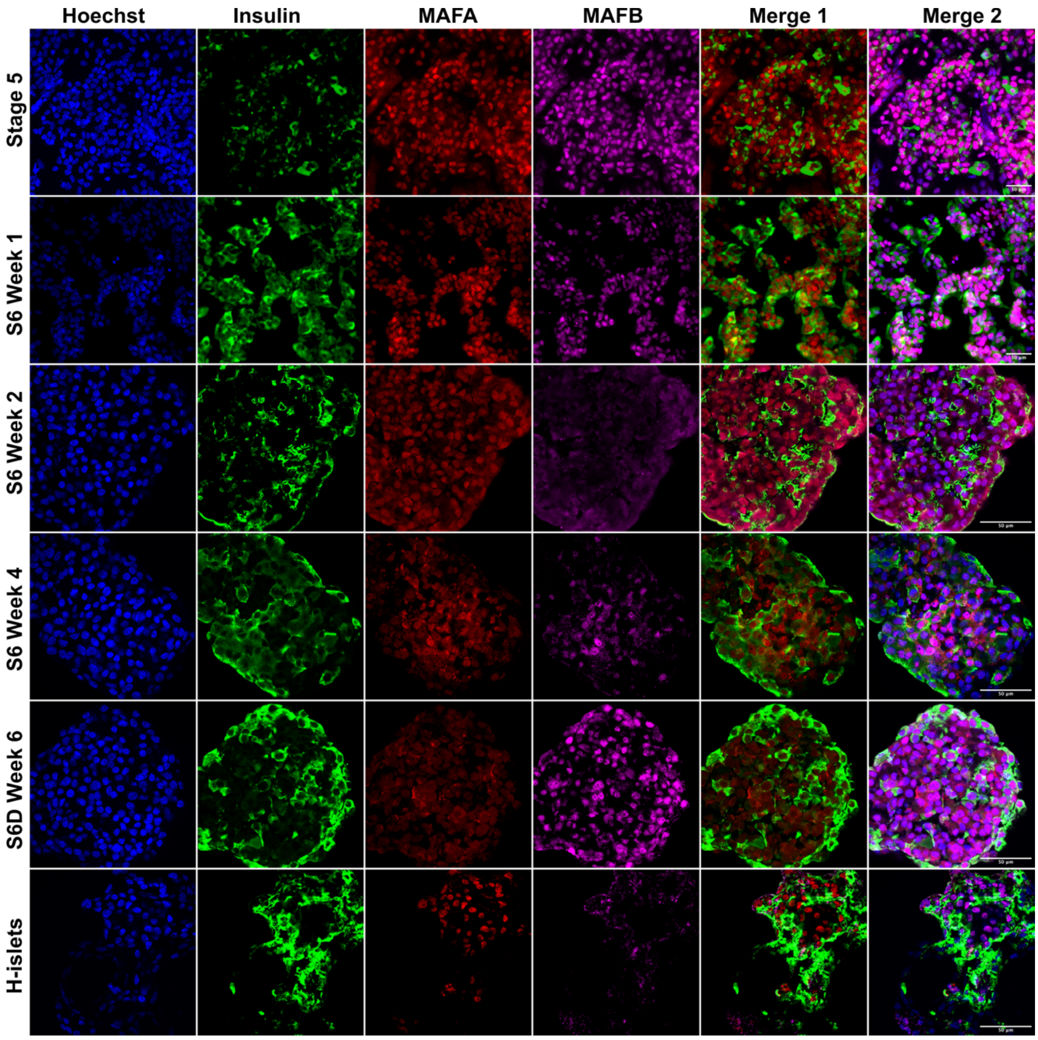

Figure S4. Immunostaining of human islets and SC-islets during prolonged culture.

Representative immunostaining sections of human islets (H-islets) and SC-islets in stage 5, week 1/2/4/6 of stage 6, for C-peptide, MAFA, MAFB. "Merge 1", Cp/MAFA; "Merge 2", Cp/MAFA/MAFB/ Nucleus. Scale bar=50 µm; Nucleus stained with Hoechst 33342.

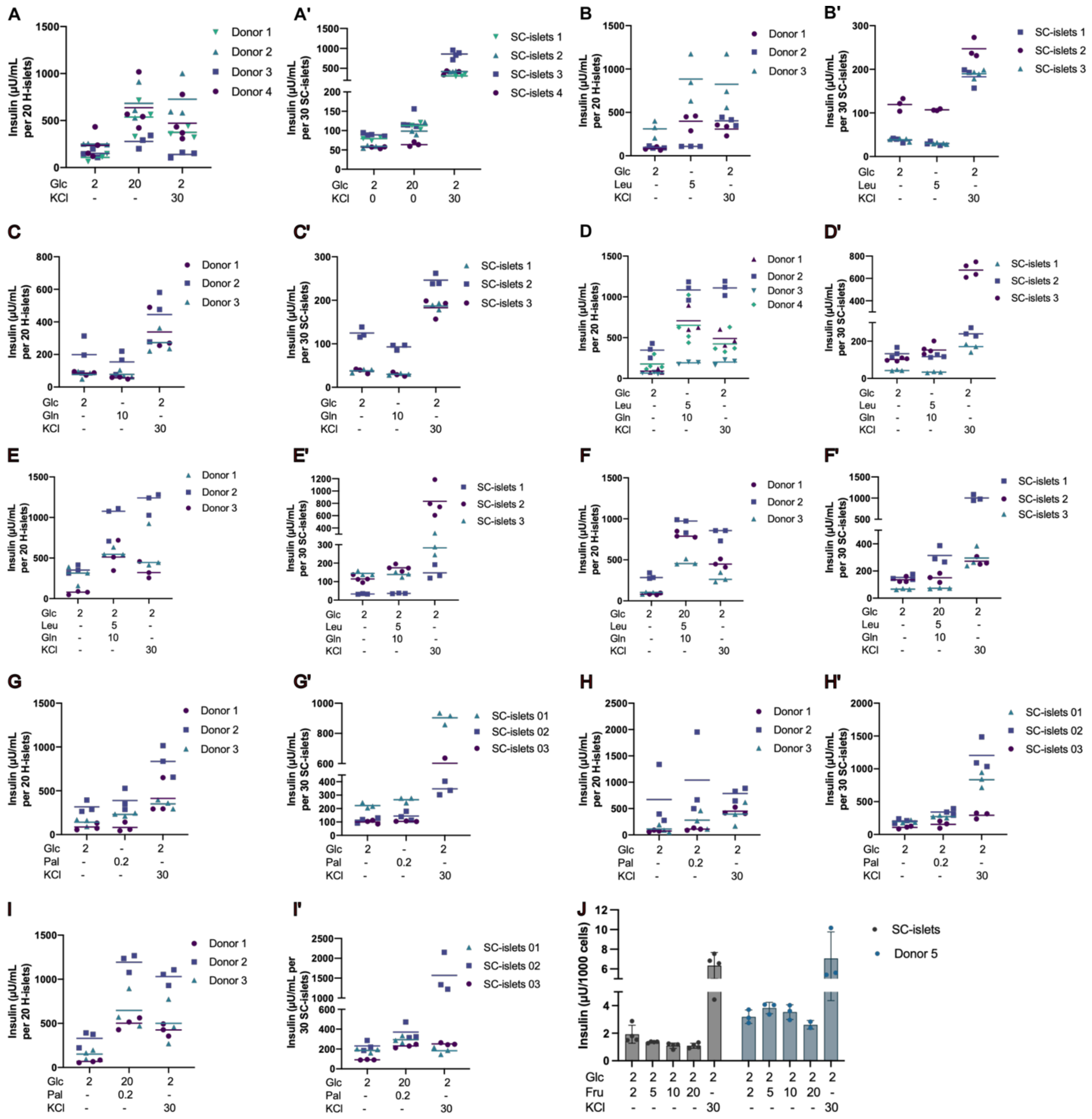

Figure S5. SC-islets and human islets' response to different nutrients.

(A and A') Human islets (A) and SC-islets (A') in response to 20 mM Glc (glucose). **(B and B')** Human islets (B) and SC-islets (B') in response to 5 mM Leu (leucine). **(C and C')** Human islets (C) and SC-islets (C') in response to 10 mM Gln (L-glutamine). **(D and D')** Human islets (D) and SC-islets (D') in response to 5 mM Leu mixed with 10 mM Gln. **(E and E')** Human islets (E) and SC-islets (E') in response to 2 mM Glc, mixed with 5 mM Leu and 10 mM Gln. **(F and F')** Human islets (F) and SC-islets (F') in response to 20 mM Glc, mixed with 5 mM Leu and 10 mM Gln. **(G and G')** Human islets (G) and SC-islets (G') in response to 0.2 mM Pal (palmitate). **(H and H')** human islets (H) and SC-islets (H') in response to 2 mM Glc, mixed with 0.2 mM Pal. **(I and I')** Human islets (I) and SC-islets (I') in response to 20 mM Glc mixed with 0.2 mM Pal. **(J)** SC-islets in response to 2/5/10/20 mM fructose (Fru). Statistical test: Two-way ANOVA with Tukey's multiple comparisons test. N=4 for SC-islets, N=2-3 for human islets.

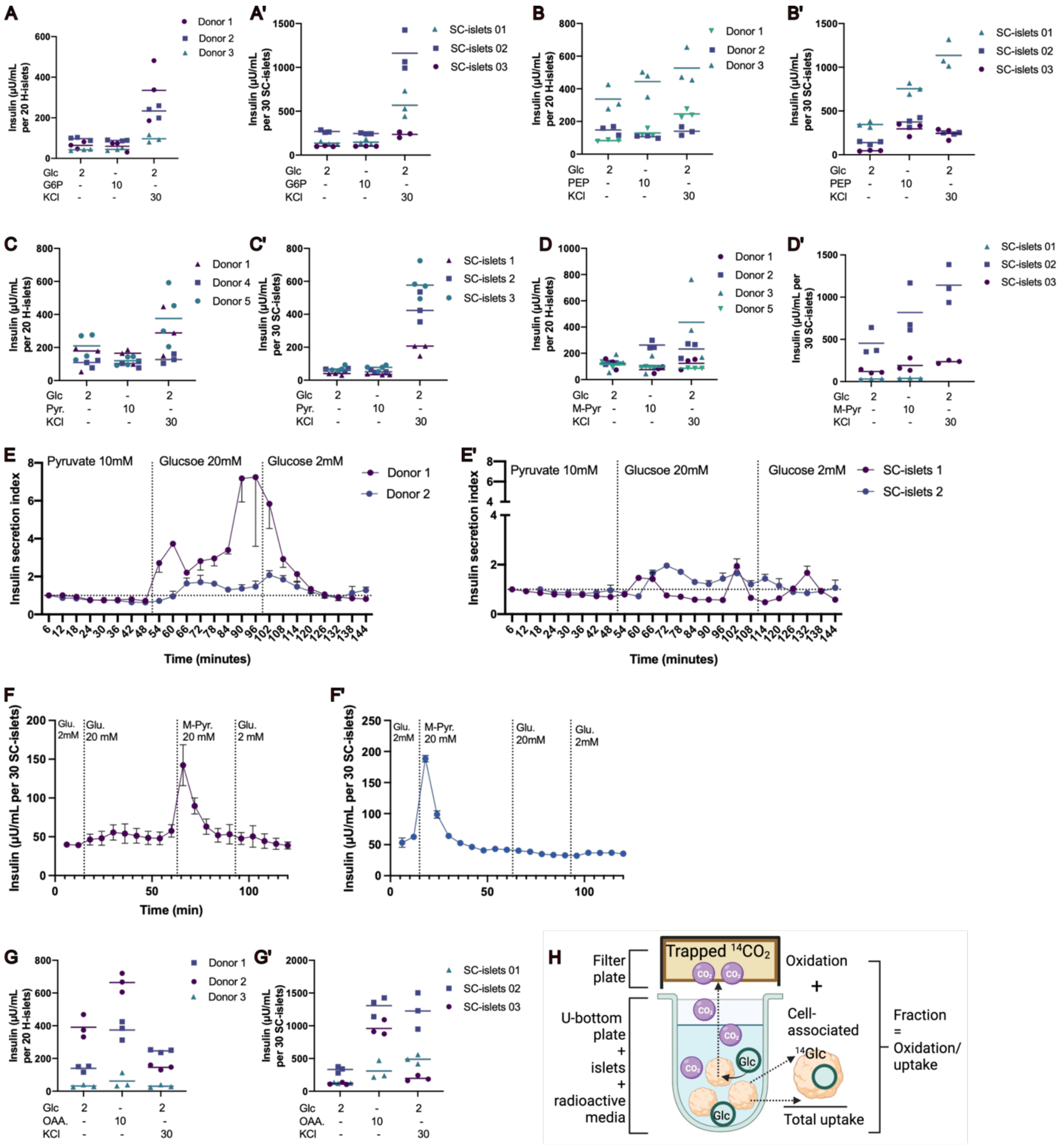

Figure S6. SC-islets and human islets' response to different inter-metabolites.

(A and A') Human islets (A) and SC-islets (A') in response to 10 mM G6P (Glucose 6-phosphate). **(B and B')** Human islets (B) and SC-islets (B') in response to 10 mM PEP (phosphoenolpyruvic acid). **(C and C')** Human islets (C) and SC-islets (C') in response to 10 mM pyruvate. **(D and D')** Human islets (D) and SC-islets (D') in response to 10 mM M-Pyr (Methyl-pyruvate). **(E and E')** Dynamic profusion for SC-islets with 2 mM Glc, 20 mM Glc, and 10 mM Pyruvate. Data presented as mean ± SEM. **(F and F')** Dynamic profusion for SC-islets with 2 mM Glc, 20 mM Glc, and 20 mM M-Pyr. Data presented as mean ± SEM. **(G and G')** Human islets (G) and SC-islets (G') in response to 10 mM OAA (oxaloacetate acid). **(H)** Schematic depicting the method for D-[^{14}C] glucose oxidative analyzing.

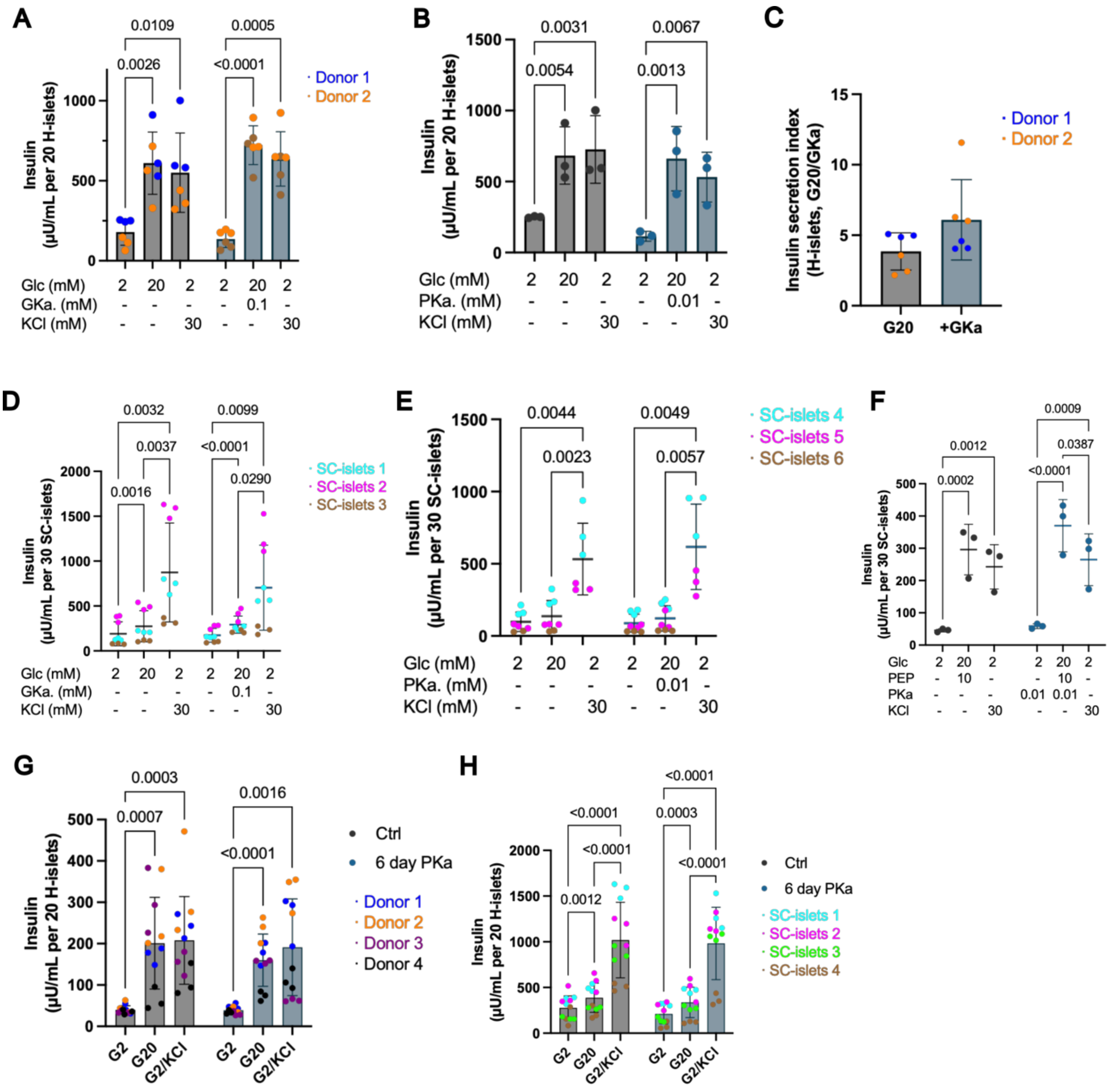

FiguFigure S7. SC-islets and human islets' response to PKa and GKa.

(A) Human islets' acute response to 0.1 mM GKa. Statistical test: Two-way ANOVA with Tukey's multiple comparisons test. n=2, N=3. **(B)** Human islets' acute response to 0.01 mM PKa. Statistical test: Two-way ANOVA with Tukey's multiple comparisons test. N=3. **(C)** Insulin secretion index of Human islets' acute response to 0.1 mM GKa. Statistical test: Mann Whitney test. n=2, N=3. **(D)** SC-islets' acute response to 0.1 mM GKa mixed with 20mM Glc. Statistical test: Two-way ANOVA with Tukey's multiple comparisons test. n=3, N=3. **(E)** SC-islets' acute response to 0.01 mM PKa mixed with 20 mM Glc. Statistical test: Two-way ANOVA with Tukey's multiple comparisons test. n=3, N=3. **(F)** SC-islets' acute response to 10mM PEP mixed with 0.01 mM PKa. Statistical test: Two-way ANOVA with Tukey's multiple comparisons test. N=3. **(G)** Insulin secretion for human islets after 6 days incubation with 0.01 mM PKa. Statistical test: Two-way ANOVA with Tukey's multiple comparisons test. n=3, N=3. **(H)** Insulin secretion for SC-islets after 6 days incubation with 0.01 mM PKa. Statistical test: Two-way ANOVA with Tukey's multiple comparisons test. n=4, N=3.

Supplementary Tables:

Supplementary Table S1: Islet donor characteristics

Islet preparation	#1	#2	#3	#4	#5	#6	#7
Unique identifier	O79	O80	O88	H2604	O121	O122	O117
Donor age (years)	49	35	57	63	31	62	25
Donor sex (M/F)[a]	F	M	M	F	M	F	M
Donor BMI (kg/m^2)	37.6	25.2	24.5	33.8	21.9	28.4	28.4
Donor HbA1c (mmol/mol)	34	N/A	N/A	31	N/A	N/A	N/A
Islet isolation centre	Oslo	Oslo	Oslo	Uppsala	Oslo	Oslo	Oslo
Donor history of diabetes?	No	No	No	No	No	No	No
For RNA Isolation and analysis	√	√	√	N/A	N/A	N/A	NA
GSIS	√	√	√	√	√	√	√

a. M, Male; F, Female.

Supplementary Table S2: List of antibodies

Antibody	Catalog No.	Vendor	Dilution	Application[1]
Rabbit anti-CHGA	NB120–15160	Novus Biologicals	1:500	IF,FC
Mouse anti-human somatostatin	sc-55565	Santa Cruz	1:600	IF,FC
Rabbit anti-human somatostatin	sc-13099	Santa Cruz	1:200	IF,FC
Rat anti-insulin/C-peptide	GN-ID4-s	University of Iowa,DHB	1:100-200	IF,FC
Mouse Anti-Glucagon	G2654	Sigma	1:800	IF,FC
Mouse anti Nkx-6.1	F55A12-S	University of Iowa,DHB	1:100	IF,FC
Goat anti Human PDX-1	AF2419	R&D Systems	1:200	IF,FC
Rabbit anti-human MAFA	ab26405	Abcam	1:100	IF
Mouse anti-human/rat MafB	MAB3810	R&D Systems	1:100	IF
Rabbit anti SLC18A1	HPA06397	Sigma	1:500	IF
Mouse anti-human CXCR4	35-8800	Invitrogen	1:500	IF
Goat anti-SOX17	AF1924	R&D Systems	1:200	FC
Rabbit Anti-HNF3b/FOXA2	07-633	Millipore	1:300	FC
Mouse anti-human CXCR4	35-8800	Invitrogen	1:200	FC

[1]IF: Immunofluorescence; WB: Western blot; FC: Flow cytometry

Supplementary Table S3: List of antibodies

**Gene name**	**Forward primer sequence**	**Reverse primer sequence**
TBP	TGTATCCACAGTGAATCTTGGTTG	GGTTCGTGGCTCTCTTATCC
INS	ACGAGGCTTCTTCTACACACC	TCCACAATGCCACGCTTCTG
GCG	CGTTCCCTTCAAGACACAGAG	GCCTGGAGTCCAGATACTTG
SST	TGGGTTCAGACAGCAGCTC	CCCAGACTCCGTCAGTTTCT
PDX1	CGTCCGCTTGTTCTCCTC	CCTTTCCCATGGATGAAGTC
NKX6.1	CTATTCGTTGGGGATGACAGAG	TGTCTCCGAGTCCTGCTTCT
ARX	CTGCTGAAACGCAAACAGAGG	CTCGGTCAAGTCCAGCCTCAT
NKX2-2	GGAGCTTGAGTCCTGAGGG	TCTACGACAGCAGCGACAAC
MAFA	GAGAGCGAGAAGTGCCAACT	TTCTCCTTGTACAGGTCCCG
MAFB	ACGCCTACAAGGTCAAGTGC	CGACTCACAGAAAGAACTCGG
ISL1	TCACGAAGTCGTTCTTGCTG	CATGCTTTGTTAGGGATGGG
UCN3	GGAGGGAAGTCCACTCTCG	TGTAGAACTTGTGGGGGAGG
NEUROD1	ATCAGCCCACTCTCGCTGTA	GCCCCAGGGTTATGAGACTAT
LDHA	GGCCTGTGCCATCAGTATCT	GGAGATCCATCATCTCTCCC
GLUT1	GCAGGCTTCTCCAACTGGAC	GAACCAGGAGCACAGTGAAG
ABCC8	GAGAAGTCGGCCTCTTTGAA	GGGCCTTTGCCATCTATACC

Supplementary Table S4: List of chemicals and reagents related to SC-islets differentiation.

**Reagents**	**Catalog No.**	**Vendor**
Penicillin-Streptomycin	15070063	Thermo Fisher Scientific
MCDB131	10372-019	Thermo Fisher Scientific
ITS-X	51500056	Gibco
Glutamax	35050061	Gibco
Fatty acid free BSA	68700	Proliant
D-Glucose	G7021	Sigma-Aldrich
Heparin	H3149	Millipore
L-Ascorbic acid	A4544	Sigma-Aldrich
Zinc sulfate heptahydrate	Z0251	Sigma-Aldrich
Sodium bicarbonate	S6297	Sigma-Aldrich
MEM NEAA	11140050	Thermo Fisher Scientific
Trace Elements A	25-021-CI	Corning
Trace Elements B	25-022-CI	Corning
Activin A	338-AC-050	R&D systems
Chir99021	71-S2924	Cellchem
IWP-2	3533	TOCRIS
KGF (FGF7)	100-19	Peprotech
LDN 193189	6053	TOCRIS
Retinoic Acid (RA)	R2625	Sigma-Aldrich
SANT1	S4572	Sigma-Aldrich
TPPB	5343	Tocris
ALK5 inh II	ALX-270-445	Enzo
Triiodothyronine (T3)	64245	Millipore
γ-secreatase inhibitor (XXi)	565789	Sigma-Aldrich
Latrunculin A	3973	TOCRIS

Supplementary Table S5: List of chemicals and reagents related to insulin secretion stimulation

Chemicals & Reagents	Abbreviations	Catalog No.	Vendor
D-Glucose	Glc	G7021	Sigma
L-Leucine	Leu	61819	Sigma
L-Glutamine	Gln	49419	Sigma
Palmitate	Pal	P5585	Sigma
Glucose-6-phosphate	G6P	10127647001	Roche
Phospho(enol)pyruvic acid	PEP	P7127	Sigma
Sodium Pyruvate	Pyruvate	11360039	ThermoFisher
Methyl Pyruvate	M-Pyr	AAA13966	VWR
Oxaloacetic acid	OAA	O4126	Sigma
Oligomycin	Oli	9996L	Cell Signaling Technology
Carbonyl cyanide 3-chlorophenylhydrazone	CCCP	C2920	Sigma
Rotenone	Rot	R8875	Sigma
Antimycin A	AA.	A8674	Sigma
UK-5099	UK.	PZ0160	Sigma
MK-0941	Gka	HY-19843	MedChemExpress
TEPP-46/ ML-265	Pka	HY-18657	MedChemExpress

Supplementary Table S6: List of abbreviations related to Figure 6G.

Name	Abbreviations	Name	Abbreviations
Glucokinase	GK	Phosphoenolpyruvate carboxykinase 2	PCK2
Pyruvate kinase	PK	Ketoisocaproic acid	KIC
Pyruvate carboxylase	PC	Glutamate dehydrogenase	GDH
Pyruvate dehydrogenase	PDH	Alpha-Ketoglutarate	a-KG
Glutamate dehydrogenase	GDH		
